# Distinctive plastome evolution in carnivorous angiosperms

**DOI:** 10.1186/s12870-023-04682-1

**Published:** 2023-12-20

**Authors:** Chao-Nan Fu, Susann Wicke, An-Dan Zhu, De-Zhu Li, Lian-Ming Gao

**Affiliations:** 1 CAS Key Laboratory for Plant Diversity and Biogeography of East Asia, Kunming Institute of Botany, Chinese Academy of Sciences, Kunming, 650201 Yunnan China; 2 Germplasm Bank of Wild Species, Kunming Institute of Botany, Chinese Academy of Sciences, Kunming, 650201 Yunnan China; 3 Lijiang Forest Biodiversity National Observation and Research Station, Kunming Institute of Botany, Chinese Academy of Sciences, Lijiang, 674100 Yunnan China; 4 Institute for Biology, Humboldt-University Berlin, Berlin, Germany; 5 Späth-Arboretum of the Humboldt-University Berlin, Berlin, Germany

**Keywords:** Carnivory, Plastome, Substitution rates, Prey-derived nutrient utilization

## Abstract

**Background:**

Independent origins of carnivory in multiple angiosperm families are fabulous examples of convergent evolution using a diverse array of life forms and habitats. Previous studies have indicated that carnivorous plants have distinct evolutionary trajectories of plastid genome (plastome) compared to their non-carnivorous relatives, yet the extent and general characteristics remain elusive.

**Results:**

We compared plastomes from 9 out of 13 carnivorous families and their non-carnivorous relatives to assess carnivory-associated evolutionary patterns. We identified inversions in all sampled Droseraceae species and four species of *Utricularia*, *Pinguicula*, *Darlingtonia* and *Triphyophyllum*. A few carnivores showed distinct shifts in inverted repeat boundaries and the overall repeat contents. Many *ndh* genes, along with some other genes, were independently lost in several carnivorous lineages. We detected significant substitution rate variations in most sampled carnivorous lineages. A significant overall substitution rate acceleration characterizes the two largest carnivorous lineages of Droseraceae and Lentibulariaceae. We also observe moderate substitution rates acceleration in many genes of *Cephalotus follicularis*, *Roridula gorgonias*, and *Drosophyllum lusitanicum*. However, only a few genes exhibit significant relaxed selection.

**Conclusion:**

Our results indicate that the carnivory of plants have different effects on plastome evolution across carnivorous lineages. The complex mechanism under carnivorous habitats may have resulted in distinctive plastome evolution with conserved plastome in the *Brocchinia hechtioides* to strongly reconfigured plastomes structures in Droseraceae. Organic carbon obtained from prey and the efficiency of utilizing prey-derived nutrients might constitute possible explanation.

**Supplementary Information:**

The online version contains supplementary material available at 10.1186/s12870-023-04682-1.

## Background

Carnivorous plants, also known as insectivorous plants, can capture and digest animal prey, absorb metabolites (nutrients) from killed prey, and utilize them for plant growth and development [1, 2]. Approximately 810 carnivorous plant species have been recognized, with several new species being described continuously and *Triantha* was the most recently described [1, 3]. Carnivory has evolved at least 13 times independently during the evolution of flowering plants with four origins in the monocot (three in Poales and one in Alismatales) and nine in eudicots (three in Caryophyllales, three in Lamiales, two in Ericales and one in Oxalidales), giving rise to 21 plant genera in 13 families of six orders [3–8]. Nearly 98% of carnivorous species belong to the four plant families Lentibulariaceae, Droseraceae, Nepenthaceae, and Sarraceniaceae, and the remaining nine families harbor only one or a few carnivorous representatives [5].

**Fig. 1:**
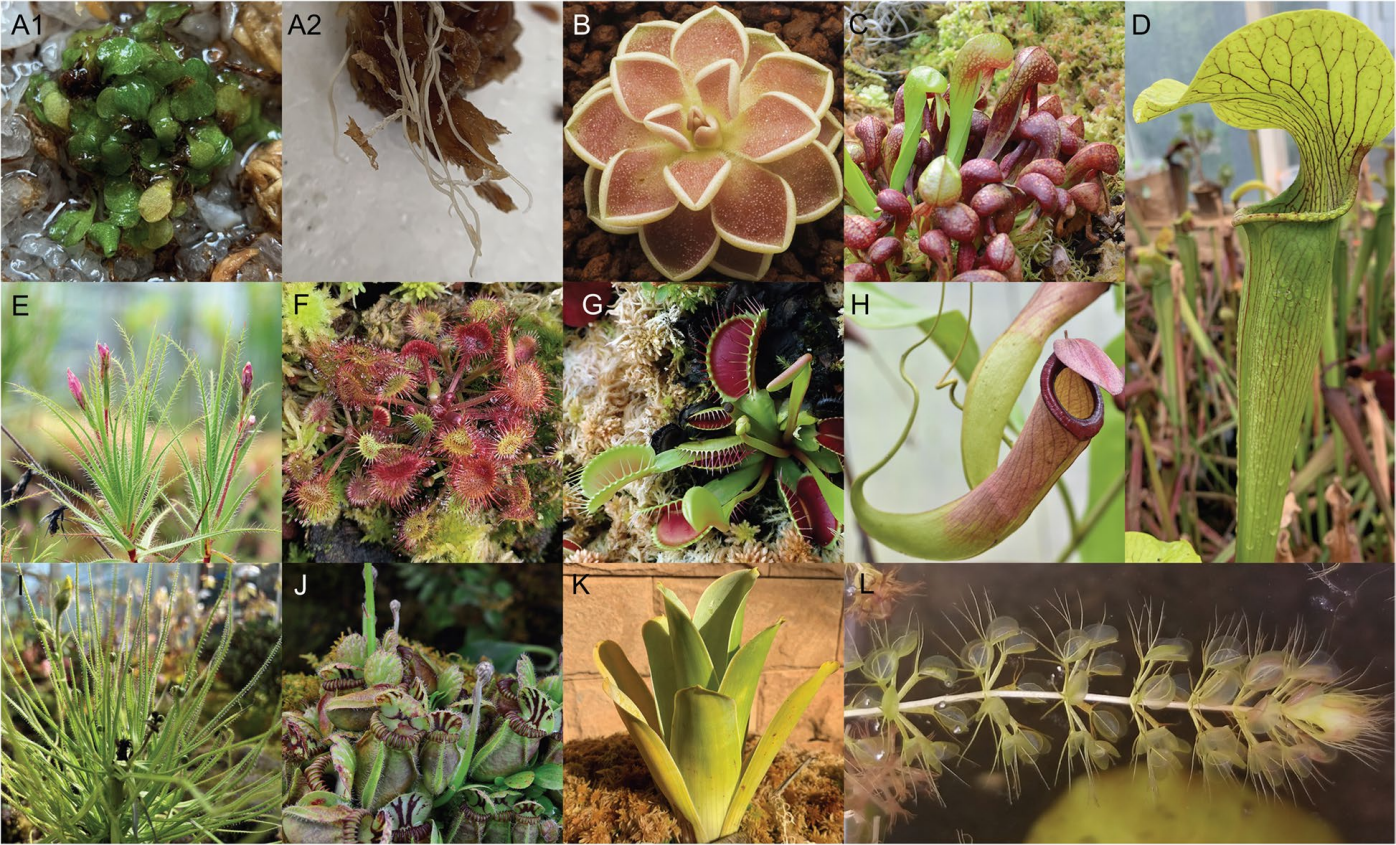
Photos of representative carnivorous plants in this study. (**A**1) *Genlisea filiformis*, (**A**2) traps of *Genlisea filiformis*, (**B)** *Pinguicula ehlersiae*, (**C)** *Darlingtonia californica*, (**D)** *Sarracenia alata*, (**E)** *Roridula gorgonias*, (**F)** *Drosera rotundifolia*, (**G)** *Dionaea muscipula*, (**H)** *Nepenthes mirabilis*, (**I)** *Drosophyllum lusitanicum*, (**J**) *Cephalotus follicularis*, (**K)** *Brocchinia hechtioides*, (**L)** *Aldrovanda vesiculosa*

Convergently evolved modified leaves allowed the carnivorous lineages to expand their functional–anatomical realm to consume prey (Fig. 1). Pitfall (“pitcher”) traps (Fig. 1 C, D, H, J, K) could capture prey in pitcher-like modified leaves that contain a pool of digestive enzymes or bacteria. This trap type has evolved convergently at least six times in Sarraceniaceae (*Sarracenia*, *Heliamphora, Darlingtonia*), Cephalotaceae (*Cephalotus*), Nepenthaceae (*Nepenthes*), Eriocaulaceae (*Paepalanthus*), and twice in Bromeliaceae (*Brocchinia* and *Catopsis*, respectively) [8]. Adhesive (“flypaper”) traps (Fig. 1 B, E, F, I) use sticky mucilage on the leaf surface in Droseraceae (*Drosera*), Drosophyllaceae (*Drosophyllum*), *Dioncophyllaceae* (*Triphyophyllum*), Lentibulariaceae (*Pinguicula*), Roridulaceae (*Roridula*), Plantaginaceae (*Philcoxia*), and a recently described genus of Tofieldiaceae (*Triantha*) [3, 8, 9]. In contrast, the snap traps (Fig. 1 G, L) in *Dionaeae* and *Aldrovanda* (Droseraceae) utilize rapid leaf movements [9]. Lentibulariaceae exhibit two unique trap types: the eel (corkscrew) traps (Fig. 1 A1, A2) of *Genlisea* and the suction traps of *Utricularia* [10]. Eel traps form inward-pointing hairs to force prey to move toward the digestive organ; suction traps suck in prey by generating an internal vacuum in bladder-like leaves [11, 12].

Carnivorous species sharing the same trap type may still vary greatly regarding the diversity of trap structures and nutrient utilization methods. For example, *Triphyophyllum* only produces carnivorous leaves for a short time before the peak of the rainy season [13]. Two species of *Roridula* with adhesive traps cannot secrete their own digestive enzymes, but absorb nitrogen from feces of symbiotically associated hemipterans that live on the plant-captured prey [14–16]. Similarly, a few members of *Nepenthes* acquire nitrogen from the feces and urine of mutualistic mammals that they attract [17–19].

Most carnivorous plants are terrestrial, with the exception of aquatic or amphibious *Aldrovanda vesiculosa* and *ca.* 60 *Utricularia* species [1]. Terrestrial carnivorous plants have high habitat specificity and grow mainly in open, infertile, and moist sites, where they can hardly absorb nutrients. They often display a low photosynthesis rate and slow growth rate compared with non-carnivorous herbs [9, 20]. In contrast, aquatic carnivorous plants mostly grow in shallow standing, oligo-mesotrophic and dystrophic waters. They exhibit much higher photosynthesis rates and growth rates than terrestrial carnivorous plants and similar rates to non-carnivorous aquatic plants [2, 20, 21]. Prey animals supplying nutrients, particularly nitrogen (N), can increase photosynthesis rate and, furthermore, may stimulate nutrient uptake from roots for growth rate increase [2, 9].

Carnivorous plants generally absorb inorganic ions and small organic molecules via selective carriers, whereas direct absorption of a mixture of organic substances, including proteins, via endocytosis was also found in some carnivorous groups [22, 23]. Although it is still debated whether or not the carnivores can directly uptake organic substances as partial substitutes for photosynthesis, recent studies have shown that amino acids absorbed from prey may serve as organic carbon sources for respiratory energy gain in *Dionaea muscipula* [24, 25], implying that carnivory possibly coincides with a partially heterotrophic or mixotrophic lifestyle in some lineages [9, 26].

Heterotrophic plants show some common characteristics in their plastome, including a small genome size, a reduced coding repertoire and noncoding region, genomic rearrangement, a high AT content, elevated substitution rate, etc. (reviewed in [27]). Distinctive photosynthesis rate and mixotrophic feeding strategy in carnivorous lineages imply that carnivorous plants may have distinct evolutionary pattern in their plastome. It was hypothesized that plastomes of carnivores might depart from the plastome stasis of most angiosperm lineages, with resemblance to the molecular-evolutionary trajectory of heterotrophic plants [26]. Corroborating this hypothesis are the observation of extraordinarily reconfigured plastomes in the Droseraceae, where multiple rearrangements, gene losses, and large expansions or contractions of the inverted repeats (IRs) occur in all the investigated species [28, 29]. For example, all *ndh* genes were lost from Droseraceae species, and some representatives also lack plastid-encoded genes like *clpP*, *ycf1*, *ycf2*, and some tRNAs. Lentibulariaceae exhibit a relatively conserved plastome structure, with the exception of massive *ndh* gene losses in all terrestrial, but not aquatic species [26, 30]. Additionally, altered proportions of repeat DNA, a significant plastome-wide increase of substitution rates and microstructural changes (indels) were observed in Lentibulariaceae plastomes [26]. In contrast, conserved plastomes with or without *ndh* genes losses characterize the plastome of *Cephalotus follicularis* [31] and *Nepenthes* × *ventrata* [29], respectively.

Changing from autotrophy to mixo- or heterotrophy is thought to have an impact on plastid genome evolution [2, 32]. Partial carnivorous nutrition may mitigate the selective pressure on plastome, resulting in a shift in substitution rate and gene loss in some lineages [26]. It is, however, unknown to date as to whether the available and taxonomically under-represented data, ranging from none to dramatic changes are associated with the varying extent of the implementation of the carnivorous syndrome in plants or due to other environmental factors. Besides, the focus on individual carnivorous species combined with the use of different analysis approaches could not track all aspects of potentially carnivorous-associated molecular-evolutionary paths. We aimed to fill this gap by broadly examining the plastomes of 28 carnivorous plants representing the majority of carnivorous families (9 out of 13) and their closely related non-carnivorous relatives across five angiosperm orders. Our phylogenomic comparative approach answers or discusses the following questions: 1) whether the nutritional benefit of carnivory impact the evolution of plastome, 2) whether the plastome evolutionary trajectories are convergent across in carnivorous plants, and 3) if the evolutionary patterns were different, what the underlying causes related to plastome variation in carnivorous plants are. Our results provide insight into the distinct evolutionary pattern of plastome across carnivorous lineages in angiosperms.

## Results

### Structural diversity of plastid genome in carnivorous plants

**Fig. 2:**
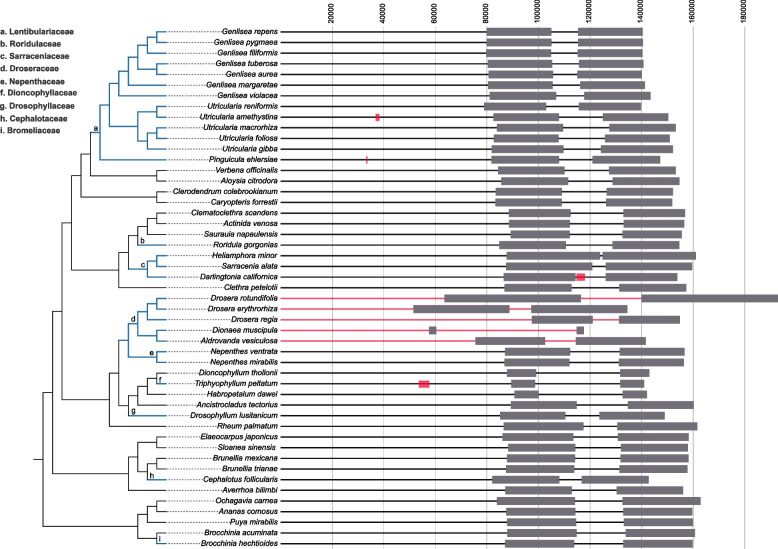
Variations in the genome structure and IR lengths of carnivorous plants and their non-carnivorous relatives. The phylogenetic relationship is constructed using all the plastid protein coding genes with all samples, and the support value for each node was shown in Figure S1. Branches leading to Carnivorous lineages (blue text) are shown with thick lines with blue color, and the families that they belong to are listed in the upper left corner with the alphabet marked in each node. Grey rectangles represent IR regions, and red rectangles represent genome rearrangements (inversions) in carnivorous species compared to non-carnivorous relatives. Red lines in Droseraceae species represent dramatic rearrangements, and a detailed rearrangement picture showed in Figure S3

The plastomes of carnivorous plants exhibit a wide range of structural diversity range from highly conservation to significant variations in genome size, IR length, gene content, genomic arrangements, and repeat structure (Supporting Table S1, Table S3). For example, plastome sizes changed substantially in Droseraceae, ranging from 117,589 bp in *Dionaea muscipula* to 192,912 bp in *Drosera rotundifolia*. Genome sizes of other carnivorous species ranged from 139,725 bp (*Utricularia reniformis*) to 161,051 bp (*Heliamphora minor*) (Fig. 2, Supporting Table S1). Compared to their non-carnivorous relatives, genome sizes have reduced in all sampled species of Droseraceae except for the dramatic expansion in *Drosera rotundifolia*, and also in all terrestrial Lentibulariaceae species, *Triphyophyllum peltatum*, *Drosophyllum lusitanicum,* and *Cephalotus follicularis*. Plastome sizes vary slightly in *Roridula gorgonias*, *Brocchinia hechtioides*, two species of *Nepenthes,* and the aquatic *Utricularia*. The lengths of IRs in Droseraceae differ more than 18-fold, ranging from 2.8 kb in *Dionaea muscipula* to ~ 52.9 kb in *Drosera rotundifolia*. An expansion of the IR region was also observed in Sarraceniaceae species, and contraction of IR region was observed in both the carnivorous and non-carnivorous Dioncophyllaceae species. Other carnivorous plants maintained a conserved typical IR region (24,101–27,905 bp). The noncoding regions were extended in Sarraceniaceae species and contracted in *Triphyophyllum peltatum* (Fig. 2, Supporting Table S1).

Drastic rearrangements of the plastid genome occurred in all sampled Droseraceae species (Fig. 2, Supporting Figure S3). Besides, a large inversion was observed in the plastomes of *Utricularia amethystine*, *Pinguicula ehlersiae*, *Darlingtonia californica*, and *Triphyophyllum peltatum* (Fig. 2, Supporting Figure S3, Supporting Table S1). The plastome structures of the remaining 22 carnivorous species were collinear with those of typical angiosperms, lacking any structural reconfigurations or gene relocations.

Carnivorous plants generally showed similar repeat content as non-carnivorous plants, yet *Drosera rotundifolia* had an extremely high number of repeats larger than 20 bp (1,089) (Supporting Figure S4, Table S1). Besides, *Triphyophyllum peltatum* (Dioncophyllaceae) showed more total repeats (340) than the sampled non-carnivorous relatives (82–205). The repeat content varied dramatically across species of Droseraceae (ranging from 72 to 1,089) and Lentibulariaceae (ranging from 44 to 215). The repeat contents of other carnivorous plants were similar to their non-carnivorous relatives or did not show consistent variation. Repeats of 50–100 bp and larger than 100 bp in length were rarely found in both the carnivorous and non-carnivorous plants, with minor departures in some species of Droseraceae and *Triphyophyllum* (Supporting Figure S4, Table S1).

## The patterns of gene losses in plastid genomes

**Fig. 3:**
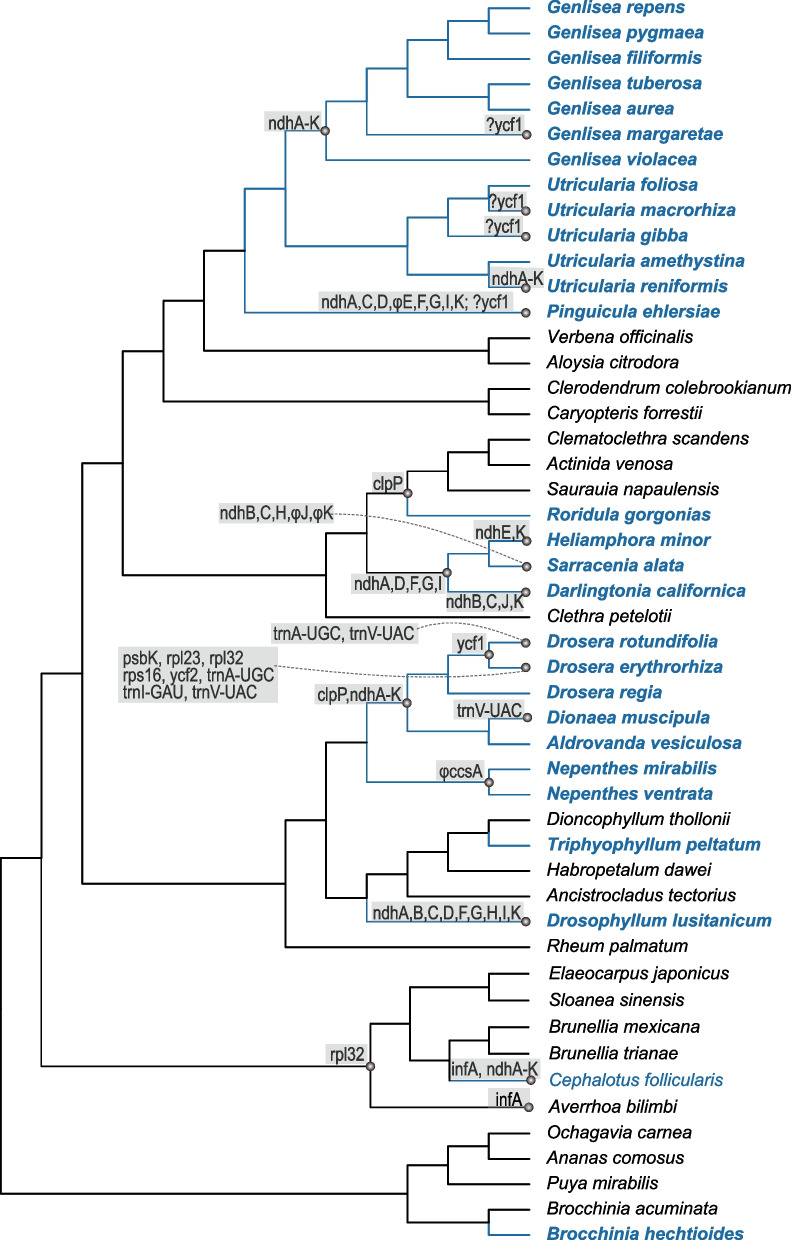
The extent of gene losses across carnivorous and non-carnivorous lineages. Branches leading to carnivorous lineages (blue text) are shown with thick lines in blue color. Commonly lost genes are list in the internal nodes; species-specific lost genes are listed at the tip nodes. The “φ” symbol before a gene name represents a pseudogene, and “?” symbol before a gene name represents an uncertain gene loss

Compared to non-carnivorous relatives, gene losses or pseudogenization have occurred in all but four sampled carnivorous species (*Brocchinia hechtioides*, *Triphyophyllum peltatum*, *Utricularia foliosa*, and *Utricularia amethystina*) (Fig. 3). In general, *ndh* genes were lost in five out of nine carnivorous lineages and other genes including *ccsA*, *clpP*, *infA*, *psbK*, *rpl23*, *rpl32*, *rps16*, *ycf1*, *ycf2*, and some tRNA genes were only independently lost in few carnivorous species. In Droseraceae, all of the eleven *ndh* genes and *clpP* have been lost. Three more genes (*trnA-UGC*, *trnV-UAC*, and *ycf1*) were lost from the plastome of *Drosera rotundifolia*, and nine more genes (*psbK*, *rpl23*, *rpl32*, *rps16*, *ycf1*, *ycf2*, *trnA-UGC*, *trnI-GAU*, and *trnV-UAC*) are absent from *Drosera erythrorhiza* plastomes; *Dionaea muscipula* has also lost the *trnV-UAC* gene. In Lentibulariaceae, all eleven *ndh* genes were lost from the plastomes of all the sampled *Genlisea* species and *Utricularia reniformis*; in *Pinguicula ehlersiae*, the *ndhA*, *C*, *D*, *E*, *F*, *G*, *I*, and *K* genes were lost or pseudogenized. The *ycf1* gene was not annotated in the published plastome of four species (*G. margaretae*, *U. macrorhiza*, *U. gibba* and *P. ehlersiae*), due to assumed sequencing technology-based errors [26]. In Sarraceniaceae, different numbers of *ndh* genes were lost in the three genera separately. The *ndhA*, *D*, *E*, *F*, *G*, *I*, and *K* genes were lost from the plastome of *Heliamphora minor*, the *ndhA*, *B*, *C*, *D*, *F*, *G*, *H*, *I*, *J*, and* K* genes were lost or pseudogenized in the plastome of *Sarracenia alata*, and *ndh A*, *B*, *C*, *D*, *F*, *G*, *I*, *J*, and* K* genes were lost in *Darlingtonia califomica*. In the plastome of *Cephalotus follicularis* (Cephalotaceae), all *ndh* genes and the *infA* gene were missing; the *rpl32* gene represents a shared loss in Cephalotaceae and its relatives Brunelliaceae and Elaeocarpaceae. In the plastome of *Drosophyllum lusitanicum* (Drosophyllaceae), nine *ndh* genes (*ndhA*, *B*, *C*, *D*, *F*, *G*, *H*, *I*, *K*) were lost. The carnivorous plant in the other four families Roridulaceae, Nepenthaceae, Dioncophyllaceae, and Bromeliaceae encode all *ndh* genes in their plastomes. However, the *clpP* gene was missing from the plastome of both *Roridula gorgonias* (Roridulaceae) and its sampled close relatives of Actinidiaceae, and *ccsA* gene is a pseudogene in the plastome of two *Nepenthes* species (Nepenthaceae) (Fig. 3, Supporting Figure S5).

## Nucleotide substitution rate variation and its association with carnivory

**Fig. 4:**
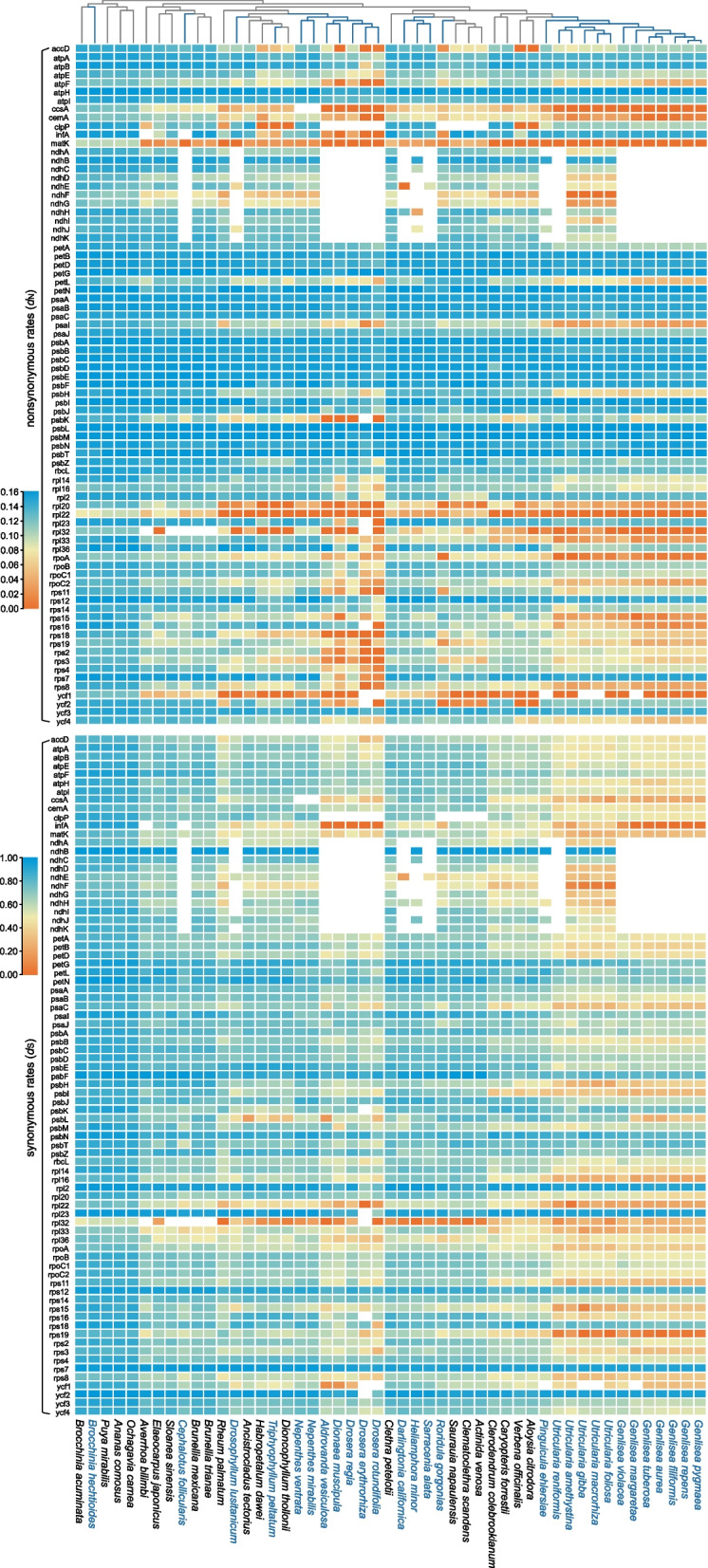
The heatmaps illustrate the rate variations in *d*_N_ (upper layer) and *d*_S_ (lower layer) for each plastid protein gene, with low rates shown in blue and high rates shown in orange

Many carnivorous lineages shown distinct but not necessarily higher substitution rates compared to their non-carnivorous relatives. The overall significant elevation was only detected in the two largest carnivorous families, Lentibulariaceae and Droseraceae (Fig. 4; Supporting Figures S6, S7, and Table S4). A moderate acceleration of synonymous substitution rate was observed in many genes of *Cephalotus follicularis*, *Roridula gorgonias*, and *Drosophyllum lusitanicum* compared with their non-carnivorous relatives (Fig. 3, Supporting Figure S6, S7). In *Triphyophyllum peltatum*, approximately half of its plastid genes have increased substitution rates than non-carnivorous relatives and half of the genes evolve slower. Carnivorous *Nepenthes* and Sarraceniaceae species have more genes with lower substitution rates than their non-carnivorous relatives, but most *ndh* genes in plastome of Sarraceniaceae show increased *d*_N_ and *d*_S_ values (Supporting Table S4).

A TraitRateProp analysis showed that substitution rate differences were associated with carnivory in many lineages (Supporting Table S5). A large number of plastid genes of Roridulaceae (63 out of 78 genes), Lentibulariaceae (55 out of 79 genes), Droseraceae (54 out of 67 genes), Nepenthaceae (46 out of 78 genes), Sarraceniaceae (46 out of 72 genes), and Drosophyllaceae (46 out of 70 genes) showed significant lifestyle-associated substitution rates change. Moderate numbers of genes with significant substitution rates change characterize Cephalotaceae (34 out of 65 genes), Bromeliaceae (32 out of 79 genes), and Dioncophyllaceae (25 out of 79 genes) plastomes. Significant differences in substitution rates occurred in both photosynthesis and housekeeping genes across all the carnivorous families alike. Substitution rates of *ndhA*, *D*, *F* and *H* genes evolved significantly different in all *ndh* genes-retained carnivorous lineages, and substitution rates of many other genes including *accD*, *atpA*, *atpB*, *ccsA*, *matK*, *petA*, *psaA*, *psaB*, *psbB*, *psbC*, *rbcL*, *rpoA*, *rpoB*, *rpoC1*, *rpoC2*, *rps3*, *ycf1* and *ycf2* genes differed significantly in all carnivorous compared with non-carnivorous relatives (Supporting Table S5).

## Changes in the selection of plastid genes

Both photosynthesis-related genes and housekeeping genes showed significant shifts in selection in some carnivorous families relative to their non-carnivorous relatives, though the patterns of selectional shifts differed across lineages. Separately, nine genes in the carnivorous lineage of Lentibulariaceae (*accD, atpE, clpP, petL, psbK, rbcL, rpoC1, ycf1, ycf2*), seven in Nepenthaceae (*atpA, cemA, clpP, psbC, rpl20, rpoB, ycf1*), six in Drosophyllaceae (*clpP, psbJ, rbcL, rpl20, rpoB, ycf2*) and Sarraceniaceae (*accD, atpA, petL, rpl22, ycf1, ycf2*), five in Droseraceae (*atpA*, *cemA, rpl14, rpoC1, ycf1*) and Roridulariaceae (*psbB, rpoB, rpoC1, rps11, ycf1*), four in Cephalotaceae (*clpP*, *petD*, *psbJ, rbcL*), and two in Bromeliaceae (*rps2, cemA*) exhibited significantly relaxed selective constraints associated with carnivory; no such shifts were detected in Dioncophyllaceae (Table 1, Supporting Table S6). The *ycf1* genes evolved under relaxed selective constraint in five out of nine carnivorous lineages, while other genes evolved under relaxed selection in a few carnivorous lineages. In contrast, many genes appear to experience intensified selection, including four (*ndhC*, *E*, *H*, *J*) out of five remaining intact *ndh* genes (*ndhB, C*, *E*, *H*, *J*) in Sarraceniaceae.

**Table 1:** genes showed significant change of ω values across carnivorous lineages in each family. The detailed information was shown in Table S5

Families with carnivorous species	Genes with relaxed selection	Genes with intensified selection
Cephalotaceae	*clpP, petD, psbJ, rbcL,*	*petA, ycf2*
Droseraceae	*atpA*, *cemA, rpl14, rpoC1, ycf1*	*accD, psbJ, psbK, rpl33, rps18*
Drosophyllaceae	*clpP, psbJ, rbcL, rpl20, rpoB, ycf2*	*atpA*, *atpB*, *atpE*, *rpl33*, *rps11*, *rps12*, *ycf1*
Nepenthaceae	*atpA, cemA, clpP, psbC, rpl20, rpoB, ycf1*	*ndhA, ndhK, rpl32, rpl33, rps19*
Dioncophyllaceae		*accD, rpl36*
Lentibulariaceae	*accD, atpE, clpP, petL, psbK, rbcL, rpoC1, ycf1, ycf2*	*atpA, infA, ndhI, psbL, rpl20, ycf4*
Roridulaceae	*psbB, rpoB, rpoC1, rps11, ycf1*	*accD, atpE, atpF, infA, ndhD, rpl16, rpl20, rpoA, rps8, ycf2, ycf3*
Sarraceniaceae	*accD, atpA, petL, rpl22, ycf1, ycf2*	*ndhC, ndhE, ndhH, ndhJ, rpoA, rps11, rps14, rps18, rps3*
Bromeliaceae	*rps2, cemA*	*rpl16*

## Discussion

### Plastome structure variations in carnivorous plants

The carnivory in angiosperms only affect the plastome structure in a few lineages and showed different patterns. Most of our sampled carnivorous lineages harbor a conserved plastome structure. Except for the dramatic rearrangements in Droseraceae species, structural reconfigurations are generally minor and species-specific in *Utricularia amethystine*, *Pinguicula ehlersiae*, *Darlingtonia californica,* and *Triphyophyllum peltatum* (Fig. 2). Elevated amounts of plastid repeat of longer than 12 bp but not longer than 50 bp were previously described in carnivorous Lentibulariaceae [26]. Considering repeats of 20 bp or longer, there are no universal differences between carnivorous and their closely non-carnivorous relatives in this study (Figure S4). Carnivorous species with inversions tend to possess more repeats larger than 50 bp or 100 bp, which may reveal a correlation between the accumulation of large repeats and genome rearrangement reported before [33, 34].

The number of genes, GC content, extension/contraction of IR region, and noncoding regions can greatly affect genome length and stability [32, 35, 36]. In carnivorous species, the variation in plastome length can be attributed to different factors. Gene content underlies plastome size variation in many lineages, including Lentibulariaceae, most Droseraceae species, as well as in *Drosophyllum lusitanicum* and *Cephalotus follicularis*. However, the extension of both IRs and noncoding regions of Sarraceniaceae species and *Drosera rotundifolia* contribute considerably more to plastome size inflation. In contrast, *Triphyophyllum peltatum*, which retains a complete set of plastid genes, exhibits a shorter plastome than its non-carnivorous relatives, mainly due to the contraction of IRs and noncoding regions (Supporting Table S1). Condensation of non-coding regions and deletion of non-essential DNA was also previously described in Lentibulariaceae plastomes, where the overall number of deletions exceeded that of insertions [26], which is also seen in parasitic plants [37].

## Plastid gene loss in carnivorous plants

The loss of plastid genes occurred independently in many carnivorous lineages. In this study, we detected gene losses or pseudogenization in all but four sampled carnivorous species (*Brocchinia hechtioides*, *Triphyophyllum peltatum*, *Utricularia foliosa*, and *Utricularia amethystina*) (Fig. 3). The loss of *ndh* genes is the most widespread, which were independently occurred in five out of nine carnivorous lineages. The loss of other genes was generally rare and independent. Only a few carnivorous species retained all *ndh* genes, including four aquatic representatives of *Utricularia*, the terrestrial species *Brocchinia hechtioides*, *Roridula gorgonias*, *Triphyophyllum peltatum*, and two *Nepenthes* species. Plastid *ndh* genes encode components of the thylakoid NAD(P)H dehydrogenase complex, which adjusts the redox level of cyclic photosynthetic electron transporters [38]. The genes were identified to be essential for plants under stress conditions [39, 40], but appears to be dispensable under favorable growth conditions, and holds limited biological significance in modern plants [41, 42]. The absence of plastid *ndh* genes were noted across photoautotrophic seed plants both in gymnosperms and angiosperms [33, 43–47], and it occurred prevalently in plants that no longer entirely rely on photosynthesis for energy and nutrients, such as parasitic and mycoheterotrophic groups. This absence represents the initial stage of plastome degradation in heterotrophic plants [27, 32, 37, 47–51]. Multiple independent losses of the *ndh* complex reveal that carnivory in plants may mitigate environmental stress or that prey-derived nutrients in some carnivorous lineages alleviate the selective pressure for the *ndh* complex in nutrient poor environment. The patterns and extent of *ndh* gene deletions may reflect the evolutionary trajectories of carnivory in angiosperms.

Except for *ndh* genes, we also found a few other photosynthesis related genes to be lost or pseudogenized, including *ccsA* in *Nepenthes* species, and *psbK*, *rpl23*, *rpl32*, *rps16* in *Drosera erythrorhiza*. In contrast, all other photosynthesis-related genes are maintained in all other carnivorous species. The preservation of the majority of photosynthesis genes corresponds to the relative normal photosynthesis capacity of carnivorous groups, but corroborates the dispensable role of the plastid *ndh* complex in these plants. Besides this, the *clpP* gene, known to be indispensable for cell survival [52], was lost in Droseraceae and *Roridula*-Actinidiaceae. This gene was also reported to be lost in other Actinidiaceae species and it might be a synapomorphy for the sister groups [53]. Loss or pseudogenization of *clpP* was also reported in some heterotrophic species like mycoheterotrophic Ericaceae [48] and parasitic *Hydnora* [54], characterizing a late stage of plastome degradation [27]. The *ycf1* gene was identified to have an essential function for chloroplasts [55]. Nonetheless, it was reported to be lost in grasses, some parasitic species, *Vaccinium*, and *Erodium* [56], and, here, in some carnivorous plants. This gene was independently lost in at least two *Drosera* species (*D. rotundifolia* and *D. erythrorhiza*), and it remains to be clarified if the gene is functional in Lentibulariaceae [26]. The *ccsA* gene is required for heme attachment to chloroplast c-type cytochromes [57], but *Nepenthes* species retain only pseudogenized copies. In addition to these three critical genes, *rpl32* was lost in *Cephalotus follicularis* and its non-carnivorous Oxalidales species. The gene *infA* is missing from the *Cephalotus follicularis* plastome. The carnivores *Drosera erythrorhiza* exhibits the most dramatic gene losses, including *psbK*, *rpl32*, *rps16*, *ycf2*, and some tRNA genes [28]. The large amount of gene loss with multiple rearrangements is similar to non-photosynthetic groups in early stages of reductive plastome evolution [27], and might be associated with the transition into an obligate carnivorous lifestyle. Gene loss has occurred in both early diverged carnivorous lineages like *Cephalotus folicularis*, *Drosophyllum lusitanicum* and Dorseraceae, as well as relative recently diverged Lentibulariaceae. In contrast, early diverged carnivorous lineages like *Roridula* and *Nepenthes* display a conserved gene content (Fig. 3, Supporting Figure S2). The extent of gene loss does not show a direct association with divergence time.

Functional intracellular transfer of *rpl32* and *infA* from the plastid to the nucleus was proven in some plants [58–60]. It is established that many photosynthesis-related plastid genes, or fragments thereof, survived in the nuclear or mitochondrial genome in some parasitic plants after their physical loss from plastomes [37]. It remains unclear whether plastid gene losses in carnivorous plants are associated with functional or nonfunctional transfers. Several losses are shared by carnivorous and closely related non-carnivorous species (*clpP*, *rpl32*), implying a functional loss or transfer in a shared ancestor. However, most gene losses occurred independently across lineages even within the same family.

## Evolution rate variation and selective regimes in plastid genes of carnivorous plants

Changes in substitution rates and selective regimes related to the carnivorous lifestyle were only evaluated in Lentibulariaceae thus far, showing that these plants exhibit elevated substitution rates in all gene classes and relaxed purifying selection in many genes [26]. Here, we detected significant substitution rate changes in many genes of most carnivorous lineages, indicating a distinct molecular evolutionary history for most carnivorous lineages compared to their non-carnivorous relatives. However, the substitution rate changes were different across lineages. We observed an overall substitution rate acceleration in carnivorous Droseraceae, Lentibulariaceae, Cephalotaceae, Roridulaceae, and Drosophyllaceae, yet not in all carnivorous lineages. The carnivorous Nepenthaceae and Sarraceniaceae also showed significant rate change, but the substitution rate acceleration was observed in part of genes. The two carnivorous plants *Brocchinia hechtioides* and *Triphyophyllum peltatum* resemble the patterns of molecular evolution of their non-carnivorous relatives (Fig. 4, Supporting Table S5).

Increased substitution rate could be a result of relaxed negative selection or positive selection on functional genes [61]. However, we did not find a direct association between relaxed selective pressure and overall substitution rate elevation in plastomes of carnivorous lineages. None to only nine genes displayed evidence of relaxed selective constraints across sampled carnivorous lineages. However, the substitution rate acceleration of certain genes like *ycf1* coincided with relaxed selective constraints in multiple carnivorous lineages, suggesting rapid evolution or dispensable role for these genes in carnivores.

Plastid genes are regulated by nuclear-encoded proteins and multi-subunit protein complexes, comprising proteins encoded by both the organelle and nuclear genome [62, 63]. Therefore, plastomes coevolve with the nuclear genome [64–66]. Altered selective constraints on nuclear genes involved in DNA replication, recombination, repair, and plastome regulation could result in shifts of plastid substitution rates. Increased nucleotide substitution rates had been observed in both plastome and multiple nuclear genes of carnivorous Lentibulariaceae species [67], later assumed to be caused by the mutagenic action of amplified reactive oxygen species (ROS) production [68].

An alternative hypothesis attributes substitution rate changes directly to the nutritional mode [26]. Substitution rate elevation was widely observed in angiosperms with heterotrophic lifestyles and in some carnivorous plants. Elevation of molecular evolutionary rates in parts of the genome may relate to relaxed selection [26]. It may also be that fully and some mixotrophic heterotrophic plants “ramp up” their metabolic rates to compensate for carbon deficits, resulting from the loss of photosynthesis, which may cause an increase in reactive free radicals and associated oxidative stress, known to be linked to increased DNA damage [69, 70]. As prey-derived organic carbon use and increased respiration rates were reported in some carnivorous plants [24, 25, 68], we hypothesize that some but not all carnivorous plants undergo a similar process as heterotrophic lineages, as discussed previously for Lentibulariaceae [26]. If that hypothesis was true, we would expect that carnivorous lineages had significantly enhanced plastome substitution rates (Lentibulariaceae, Droseraceae, *Cephalotus follicularis*, *Roridula gorgonias*, and *Drosophyllum lusitanicum*) may use prey-derived organic carbon to a greater extent than others, while other carnivorous lineages might absorb mainly inorganic ions. Interestingly, the substitution rate of many remaining *ndh* genes, *accD*, *atpA*, *atpB*, *ccsA*, *matK*, *petA*, *psaA*, *psaB*, *psbB*, *psbC*, *rbcL*, *rpoA*, *rpoB*, *rpoC1*, *rpoC2*, *rps3*, *ycf1* and *ycf2* genes were significantly different in all carnivorous lineages compared to non-carnivorous relatives, revealing a specific correspondence of these genes to the transition to carnivorous. In addition, generation time can also be linked to the substitution rate, and perennial plants usually evolve slowly than annuals, but we did not observed this pattern in the study [71].

## Potential influence of carnivory on plastid genome variation

The carnivorous plants sampled here represent the majority of carnivorous lineages in angiosperms. Our sampling included all trap types as well as representatives from both aquatic and terrestrial habitats. Our findings revealed that the plastome evolution of carnivorous plants was not directly associated with their trap types and habitats. For example, among the five sampled carnivorous genera with adhesive traps, *Drosera* has an extremely diverse plastome with multiple gene losses and rearrangements; *Drosophyllum* and *Pinguicula* have lost many plastid *ndh* genes; *Triphyophyllum* comprises all plastid genes, but the plastome has a large inversion; and *Roridula* has a conserved plastome structure and gene content. The mechanisms of prey capture, prey digestion, nutrient utilization, etc. are very complex, even when traps resemblance is high. For instance, the tentacles of *Roridula* only produce resin to capture insects, but they cannot secrete their own enzyme to digest prey [72]. Instead, the trapped insects are made consumable for the carnivorous plants by symbiotic hemipterans [14, 73], a mutualism not detected in other carnivorous plants with adhesive traps.

The plastomes of parasitic or mycoheterotrophic plants usually show convergent genome degradation, with massive rearrangements as a consequence of the relaxation of functional constraints on photosynthesis [27]. The reduction or variation occurs in a stepwise manner that concurs with the transition to an obligate heterotrophic lifestyle [32]. However, the plastomes of carnivorous plants show great variation across lineages with conserved plastome in the *Brocchinia hechtioides* to strongly reconfigured plastomes structures in Droseraceae. This may hint at different strategies and efficiencies to utilize prey-derived nutrients in the various carnivorous lineages. Carnivores with dramatic plastome variation may transit to mixotrophy which make use of larger amounts of organic carbon from prey, whereas carnivorous lineages with conserved plastome structures may rather obtain and metabolize minerals, not carbon, from their prey. Recent studies in *Dionaea muscipula* have found that not only nitrogen, but also glutamine-based carbon is absorbed and used in traps, and that the energy expenditure of the examined species partly relies on the catabolism of prey-derived organic carbon [24, 25]. This physiological ability is matched with a remarkable rearrangement, gene losses, substitution rate acceleration, and an increased repeat content in the species’ plastome. This character may have evolved in the common ancestor of Droseraceae, including the aquatic carnivorous species of *Aldrovanda*, as all species in the family exhibit dramatic plastome rearrangements. Other aquatic carnivorous species of *Utricularia* were reported to allocate far more biomass in vegetative organs, which contributed to increased photosynthetic rates by well-developed leaves [74]. These species may be autotrophic with conserved plastome. However, terrestrial species of *Utricularia* characterized by numerous losses of *ndh* genes and an accelerated substitution rate, have been reported to only allocate little biomass to vegetative parts in some species and might not be fully autotrophic [74, 75]. Other lineages with relatively conserved plastomes include *Nepenthes* and *Brocchinia* with many normal photosynthetic leaves without traps, as well as *Roridula* with mutualism of hemipterans [16] and *Brocchinia* which may have a mutualistic relationship with frog [76], and *Triphyophyllum,* which the largest part of its life being non-carnivorous [13]. These species are likely to have regular photosynthetic ability.

Since there are only a few ecophysiological experiments focusing on the fate of carbon resources from prey-derived nutrients in carnivorous plants, it is currently not possible to analyze the correlation between the plastome variation and carbon utilization in carnivorous plants. If ours and a previous hypothesis [26] on the connection between the carnivorous lifestyle and a mixotrophic carbon gain is correct, we can expect that carnivorous species with gene losses, structural modifications, and elevated molecular evolutionary rates make use of prey-derived organic carbon, whereas lineages with conserved plastomes and plastid gene content mainly utilize mineral nutrients rather than organic molecules. This means, although carnivorous plants display convergent evolution in their behaviour and/or morphology, the underlying mechanism is highly intricate, and has varying effects on plastome evolution.

## Materials and methods

### Taxa sampling, DNA extraction, and sequencing

Combining the sequences with 20 previously published plastomes, we obtained the plastome from 15 genera of nine families and five orders of carnivorous plants, representing all the carnivorous trap types and species-rich families (Supporting Table S1). Among them, eight plastid genomes (plastomes) of carnivorous plants were newly sequenced, increasing data from four independent carnivorous lineages (Sarraceniaceae, Roridulaceae, Cephalotaceae, and Bromeliaceae). To explore the evolutionary patterns of the plastid genome of carnivorous plants, we compiled a total of 48 species by also sampling closely non-carnivorous relatives for each of the carnivorous lineages, thereby achieving a total of nine pairs of carnivorous-non-carnivorous clades. For each of the carnivorous and non-carnivorous clades, we included at least five species for the selective pressure analysis (Supporting Figure S1). Newly obtained voucher specimens were collected for each species. Comprehensive information, including the individual who undertook the formal identification of the plant material, can be found in Table S1.

For newly sequenced species, total genomic DNA was extracted from fresh leaf tissue using a modified CTAB method [77]. Total DNA was quantified using Qubit 2.0 (Invitrogen, Carlsbad, CA, USA) and sheared into approximately 600 bp fragments using a Covaris M22 focused ultrasonicator. Library construction using the NEB Next Ultra II DNA Library Prep Kit for Illumina according to the standard protocol of the manufacturer. All libraries were subsequently sequenced from both ends in 150 bp mode on the Illumina HiSeq X Ten platform in CloudHealth Company in Shanghai, China to generate approximately 2 Gb clean data per sample.

## Plastome assembly, annotation and structural analysis

The newly sequenced plastomes were assembled using the GetOrganelle Toolkit [78] from cleaned sequencing data. Firstly, the plastome-associated reads were extracted from total genomic reads using a modified “baiting and iterative mapping” with Bowtie2 [79] and BLAST + [80] based on reference database plugin the toolkit. Subsequently, the potential plastome-derived reads were de novo-assembled using *SPAdes* version 3.6.2 [81]. The analysis was performed at the iFlora High-Performance Computing Center of Germplasm Bank of Wild Species (iFlora HPC Center of GBOWS, KIB, CAS). Both the newly obtained and published plastome sequences were annotated with PGA [82], with manual correction and modification in Geneious v9.0.2 (Biomatters Limited). Pseudogenes were defined based on the loss of parts in their sequences or by the presence of internal stop codons, which would be caused by nucleotide mutation, frameshift mutation, premature stop codon, etc. [37]. The collinearity of plastomes between carnivorous plants and their close relatives was assessed using the Mauve v. 2.3.1 [83, 84] plugin in Geneious. The genome size, length of the large and small single-copy regions (LSC, SSC) and inverted repeat regions (IRs), gene numbers, and GC content of each species were summarized using Geneious. Direct and palindromic long repeats were detected using REPuter (https://bibiserv.cebitec.uni-bielefeld.de/reputer; [85]), with a minimal repeat size of 20 bp and a Hamming distance of 1.

## Substitution rate and selection pressure analyses

The protein coding genes were extracted using PhyloSuite v1.2.2 [86]. Each gene was aligned using MAFFT v7.22 [87] plugin in PhyloSuite with G-INS-I algorithm and “translation align” option selected. All the genes were concatenated in PhyloSuite to generate a supermatrix.

To test for changes in substitution rates and selection pressure, a phylogenetic tree including all taxa as well as nine subtrees of carnivorous and non-carnivorous pairs were constructed using RAxML v8.1.11 [88, 89]. Tree reconstruction was carried out with the GTR + GAMMA model based on a concatenated supermatrix of all plastid protein coding genes with all taxa and nine subsets (A total tree and nine subtrees of carnivorous and non-carnivorous pair clade shown in Supporting Figure S1) separately.

The nonsynonymous rates (*d*_N_) and synonymous rates (*d*_S_) were estimated for each gene using PAML v. 4.7 [90] in codeml mode, with branch models (model = 1, *free-ratios* model) based on each gene matrix and the total tree. To compare substitution rate in the carnivorous and non-carnivorous lineages, the tip-to-root branch length for each species was calculated using software Newick Utilities [91].

The *TraitRateProp* method [92, 93] was used to test for associations between carnivory and substitution rates for each protein-coding gene of our nine carnivorous and non-carnivorous pairs set. A time tree including all samples was estimated using penalized likelihood method using treePL software [94] with ten secondary calibrations according to previous publications (Supporting Table S2). Nine dated subtrees were extracted from the whole time-dated tree (Supporting Figure S2) for the later analyses. Carnivory was coded as a binary trait (1 = carnivorous plant; 0 = non-carnivorous plant). The method was used to test the hypothesis of a trait-rate association against a null model of no association via stochastic mapping.

The RELAX model in HyPhy v 2.5.36 [95, 96] was used to test the hypothesis of relaxed and intensified selective constrains in carnivorous lineages versus their non-carnivorous relatives. The carnivorous lineage was set as “test” branches, and their most closely related non-carnivorous lineage were set as “reference” branches for each carnivorous and non-carnivorous subtree (Supporting Figure S1). The method compared two models, with a null model of same ω distribution on test and reference branches against an alternative model in which branches are allowed to have different ω distribution in a likelihood ratio test (LRT). The analyses were run via command line on a local server, and resulting ‘.json’ files were parsed with a custom python script.

### Supplementary Information

**Additional file 1: Figure S1.** The (A) total tree and (B) subtrees of nine pairs of carnivorous and non-carnivorous clades. The phylogenetic relationship is constructed using all the plastid protein coding genes. The support value for each node was shown in the total tree. Branches leading to carnivorous lineages (blue text) are shown with thick lines with blue color, and the closest non-carnivorous lineages in subtrees are shown with thick lines with orange color. **Figure S2.** Divergence time estimation of total tree with all samples. Branches leading to carnivorous lineages (blue text) are shown with thick lines with blue color. **Figure S3.** Mauve plot showing inversions in (A) Droseraceae, (B) *Utricularia amethystine*, (C) *Pinguicula ehlersiae*, (D) *Darlingtonia califonica*, and (E) *Triphyophyllum peltatum* compared to their non-carnivorous relatives. The blocks with the same color represent the genome region with similar nucleotide sequence and the blocks with same color but opposite orientation represent the genome region with inversion.**Figure S4.** Repeats content in carnivorous and non-carnivorous lineages. (A) The histogram shows the repeats content variation across carnivorous lineages and their non-carnivorous relatives. (B) Boxplot shows the difference in repeats content between carnivorous and non-carnivorous species. **Figure S5.** Gene content for each species. The black square means the gene is present in the species, the grey square means the gene is pseudogenized in the species, and the white square means the gene is absent from the species. **Figure S6.** The boxplot illustrates the difference in *d*N values between carnivorous and non-carnivorous species for each gene group of each carnivorous and non-carnivorous pair. The PS represents other photosynthesis genes, and HK represents other housekeeping genes. The “*” symbol represents *P* < 0.05, “**” represents *P* < 0.01, “***” represents *P* < 0.001, and “****” represents *P* < 0.0001. **Figure S7.** The boxplot illustrates the difference in *d*S values between carnivorous and non-carnivorous species for each gene groups of each carnivorous and non-carnivorous pair. The PS represents other photosynthesis genes, and HK represents other housekeeping genes. The “*” symbol represents *P* < 0.05, “**” represents *P* < 0.01, “***” represents *P* < 0.001, and “****” represents *P* < 0.0001.

** Additional file 2:** **Table S1**. Information of sampled species and plastome characters of carnivorous speceis and their closely related non-carnivorous species. **Table S2.** The calibration nodes used in divergence time analyses. **Table S3.** The gene content of sampled species. **Table S4.** The nonsynonymous and synonymous substitution rates of sampled species.**Table S5.** Substitution rate change test for each protein-coding gene of nine carnivorous and non-carnivorous pairs.**Table S6**. Results of RELAX (HyPhy) analyses.

## Data Availability

All DNA sequences have been deposited in the NCBI GenBank database (accession numbers in Table S1).
