# Supplementary material for: Distinctive plastome evolution in carnivorous angiosperms
Source: BMC Plant Biol. 2023 Dec 20;23:660. doi: 10.1186/s12870-023-04682-1 (PMC10731798; doi:10.1186/s12870-023-04682-1)
Supplement: Supplementary file 1 — Additional file 1: Figure S1. The (A) total tree and (B) subtrees of nine pairs of carnivorous and non-carnivorous clades. The phylogenetic relationship is constructed using all the plastid protein coding genes. The support value for each node was shown in the total tree. Branches leading to carnivorous lineages (blue text) are shown with thick lines with blue color, and the closest non-carnivorous lineages in subtrees are shown with thick lines with orange color. Figure S2. Divergence time estimation of total tree with all samples. Branches leading to carnivorous lineages (blue text) are shown with thick lines with blue color. Figure S3. Mauve plot showing inversions in (A) Droseraceae, (B) Utricularia amethystine, (C) Pinguicula ehlersiae, (D) Darlingtonia califonica, and (E) Triphyophyllum peltatum compared to their non-carnivorous relatives. The blocks with the same color represent the genome region with similar nucleotide sequence and the blocks with same color but opposite orientation represent the genome region with inversion.Figure S4. Repeats content in carnivorous and non-carnivorous lineages. (A) The histogram shows the repeats content variation across carnivorous lineages and their non-carnivorous relatives. (B) Boxplot shows the difference in repeats content between carnivorous and non-carnivorous species. Figure S5. Gene content for each species. The black square means the gene is present in the species, the grey square means the gene is pseudogenized in the species, and the white square means the gene is absent from the species. Figure S6. The boxplot illustrates the difference in dN values between carnivorous and non-carnivorous species for each gene group of each carnivorous and non-carnivorous pair. The PS represents other photosynthesis genes, and HK represents other housekeeping genes. The “*” symbol represents P < 0.05, “**” represents P < 0.01, “***” represents P < 0.001, and “****” represents P < 0.0001. Figure S7. The boxplot illustrates the [file 12870_2023_4682_MOESM1_ESM.pdf]

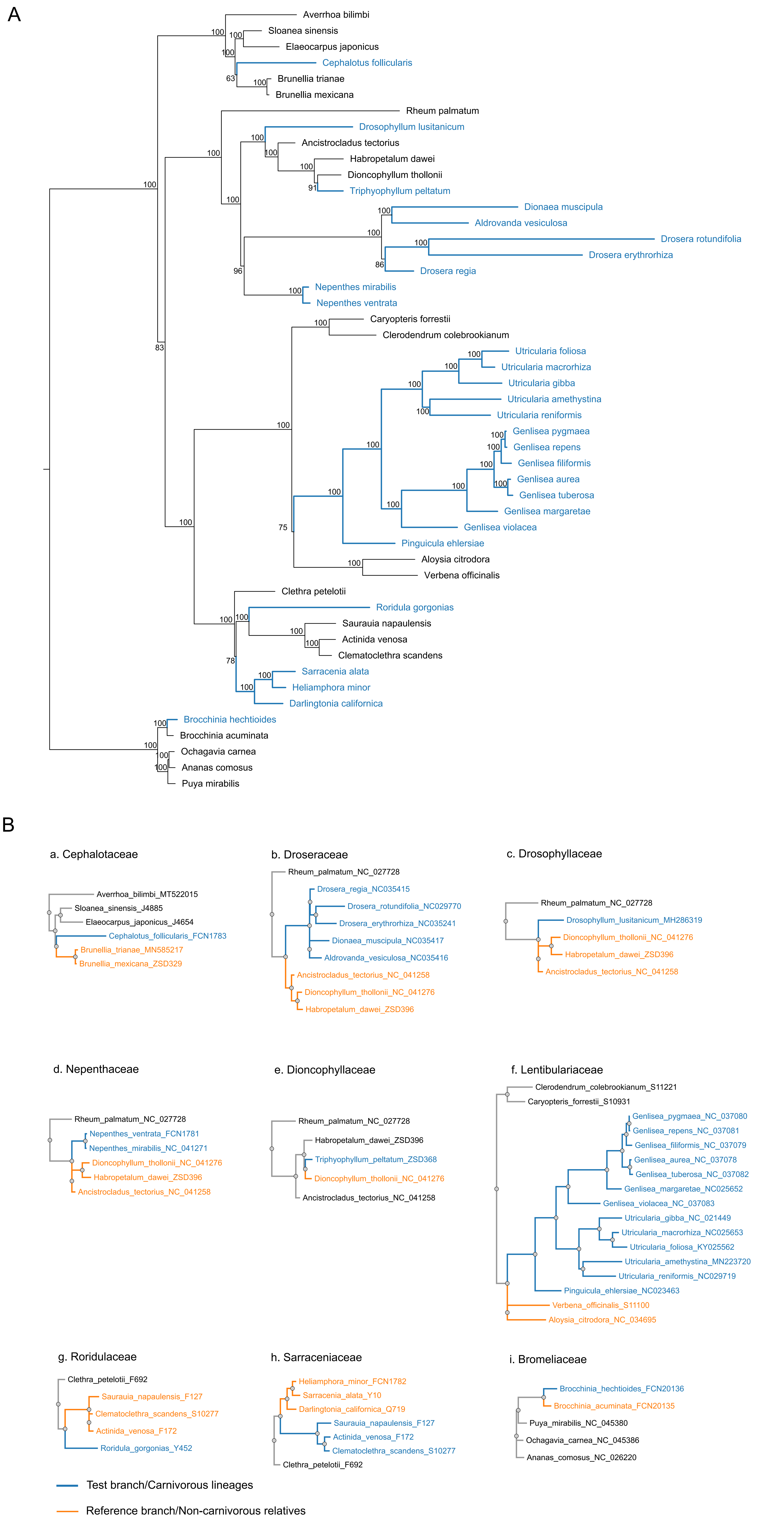

Figure S1. The (A) total tree and (B) subtrees of nine pairs of carnivorous and non-carnivorous clades. The phylogenetic relationship is constructed using all the plastid protein coding genes. The support value for each node was shown in the total tree. Branches leading to carnivorous lineages (blue text) are shown with thick lines with blue color, and the closest non-carnivorous lineages in subtrees are shown with thick lines with orange color.

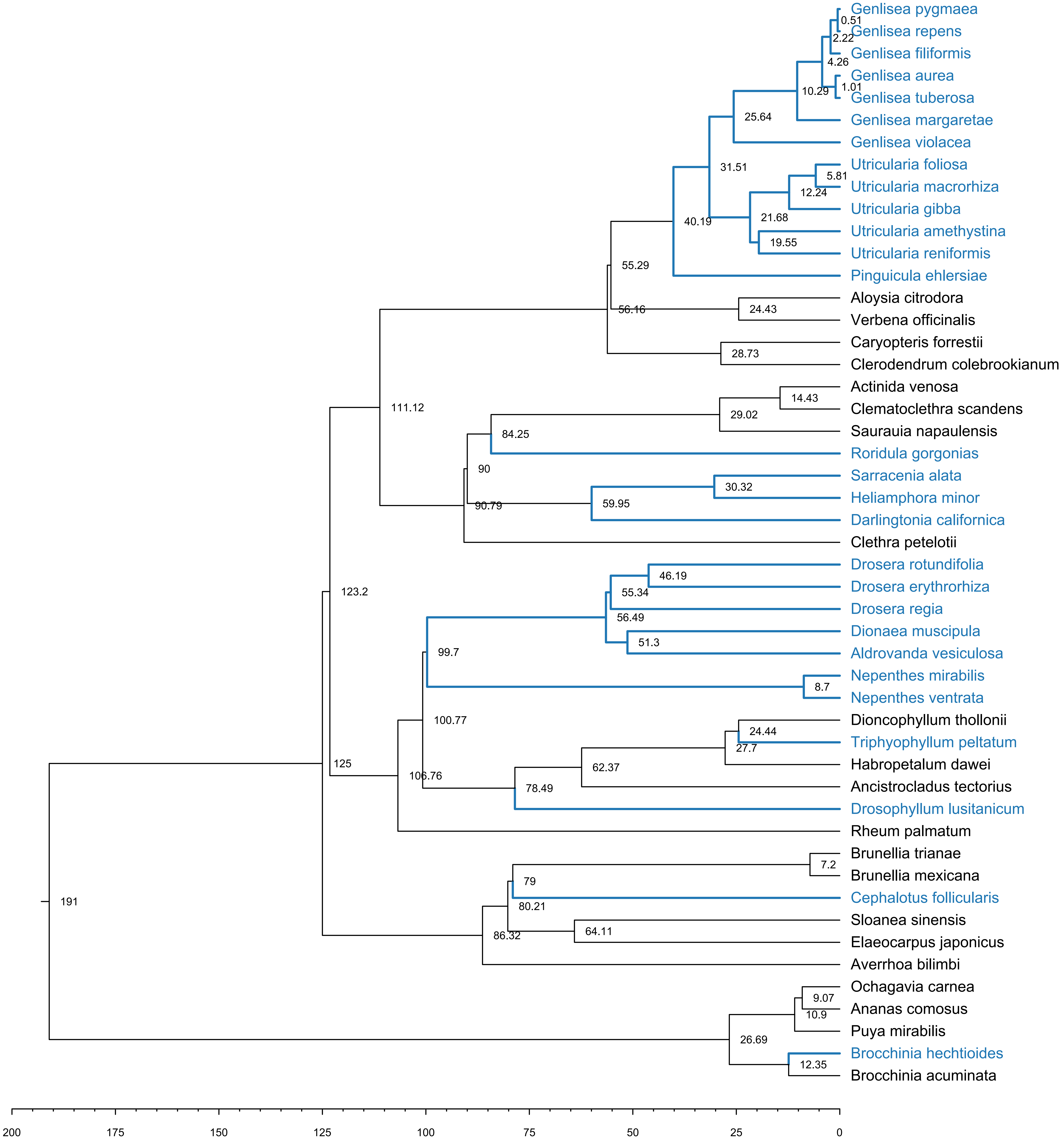

Figure S2. Divergence time estimation of total tree with all samples. Branches leading to carnivorous lineages (blue text) are shown with thick lines with blue color.

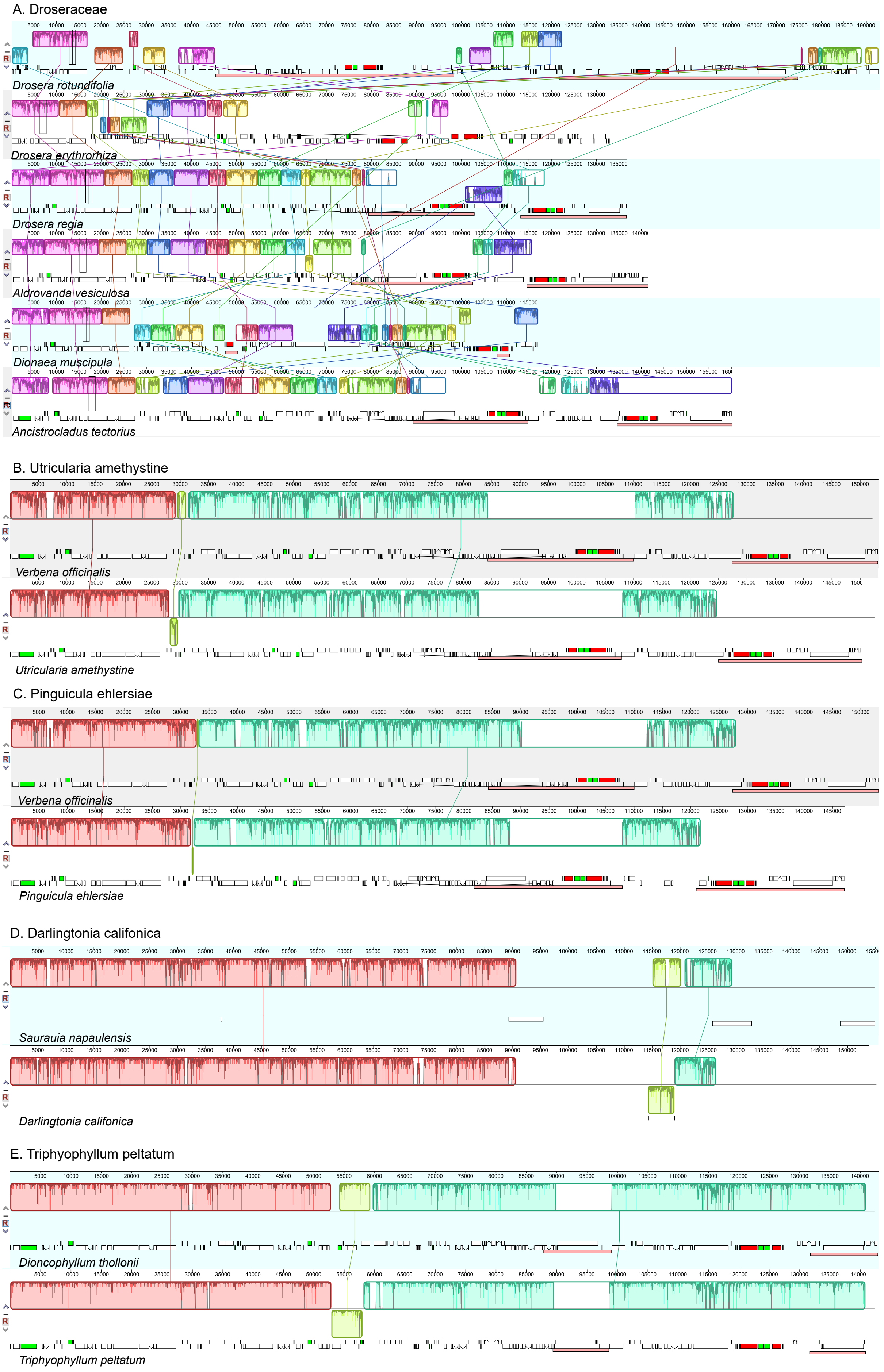

Figure S3. Mauve plot showing inversions in (A) Droseraceae, (B) *Utricularia amethystine*, (C) *Pinguicula ehlersiae*, (D) *Darlingtonia califonica*, and (E) *Triphyophyllum peltatum* compared to their non-carnivorous relatives. The blocks with the same color represent the genome region with similar nucleotide sequence and the blocks with same color but opposite orientation represent the genome region with inversion.

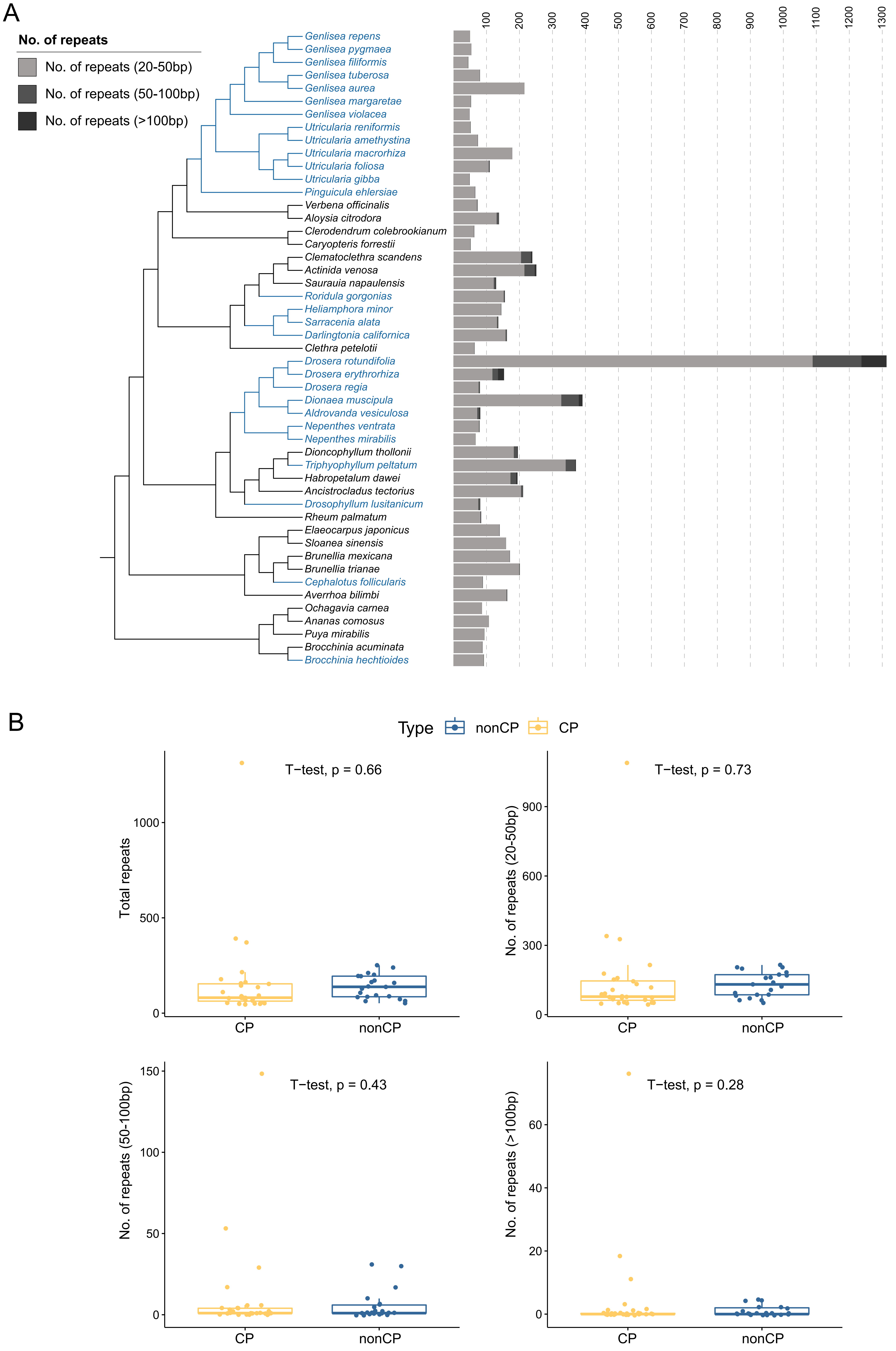

Figure S4. Repeats content in carnivorous and non-carnivorous lineages. (A) The histogram shows the repeats content variation across carnivorous lineages and their non-carnivorous relatives. (B) Boxplot shows the difference in repeats content between carnivorous and non-carnivorous species.

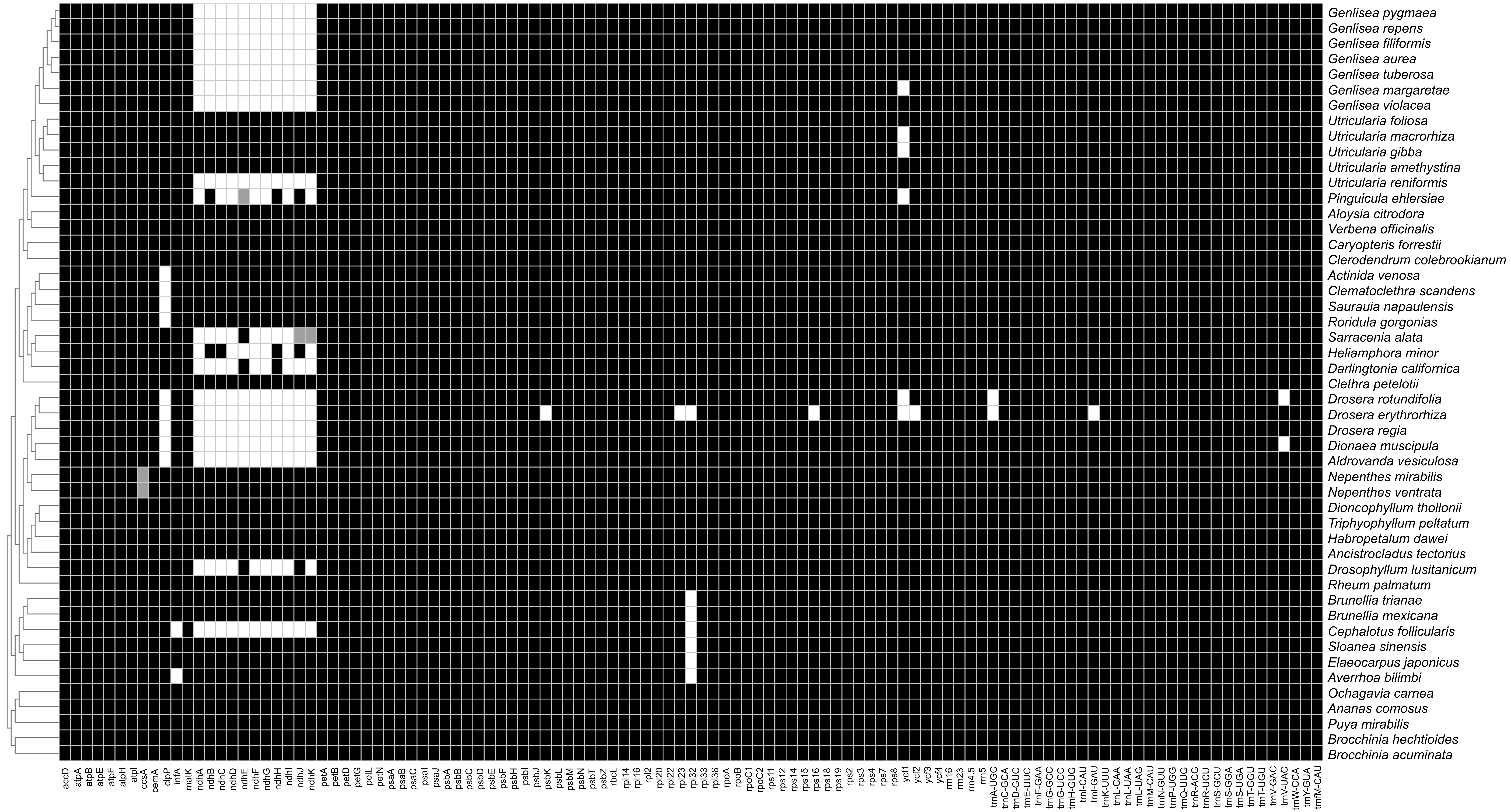

Figure S5. Gene content for each species. The black square means the gene is present in the species, the grey square means the gene is pseudogenized in the species, and the white square means the gene is absent from the species.

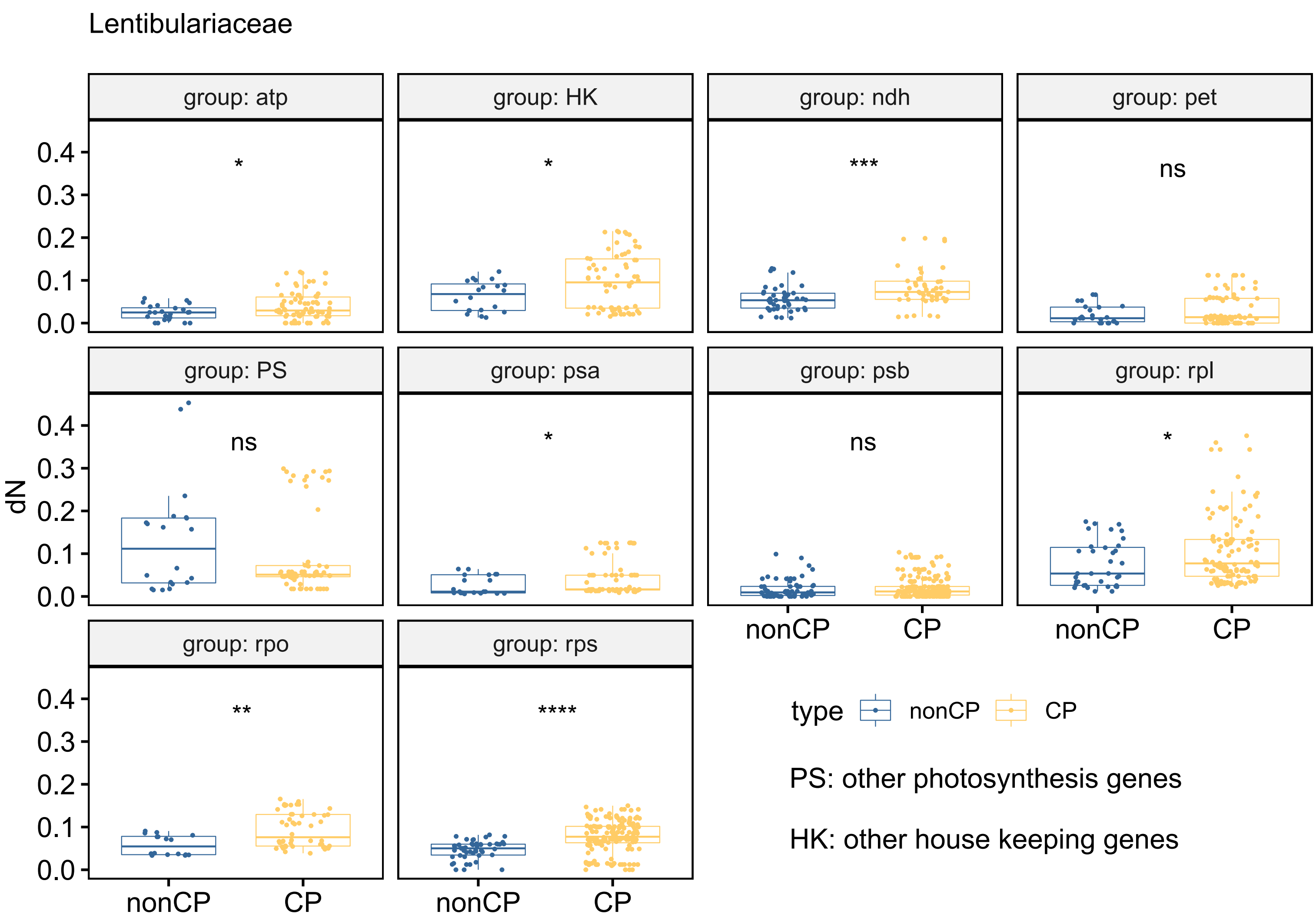
Lentibulariaceae
group: atp
group: HK
group: ndh
group: pet
group: PS
group: psa
group: psb
group: rpl
group: rpo
group: rps
*
*
***
ns
ns
*
ns
*
**
****
dN
0.0
0.1
0.2
0.3
0.4
nonCP
CP
type
nonCP
CP
PS: other photosynthesis genes
HK: other house keeping genes

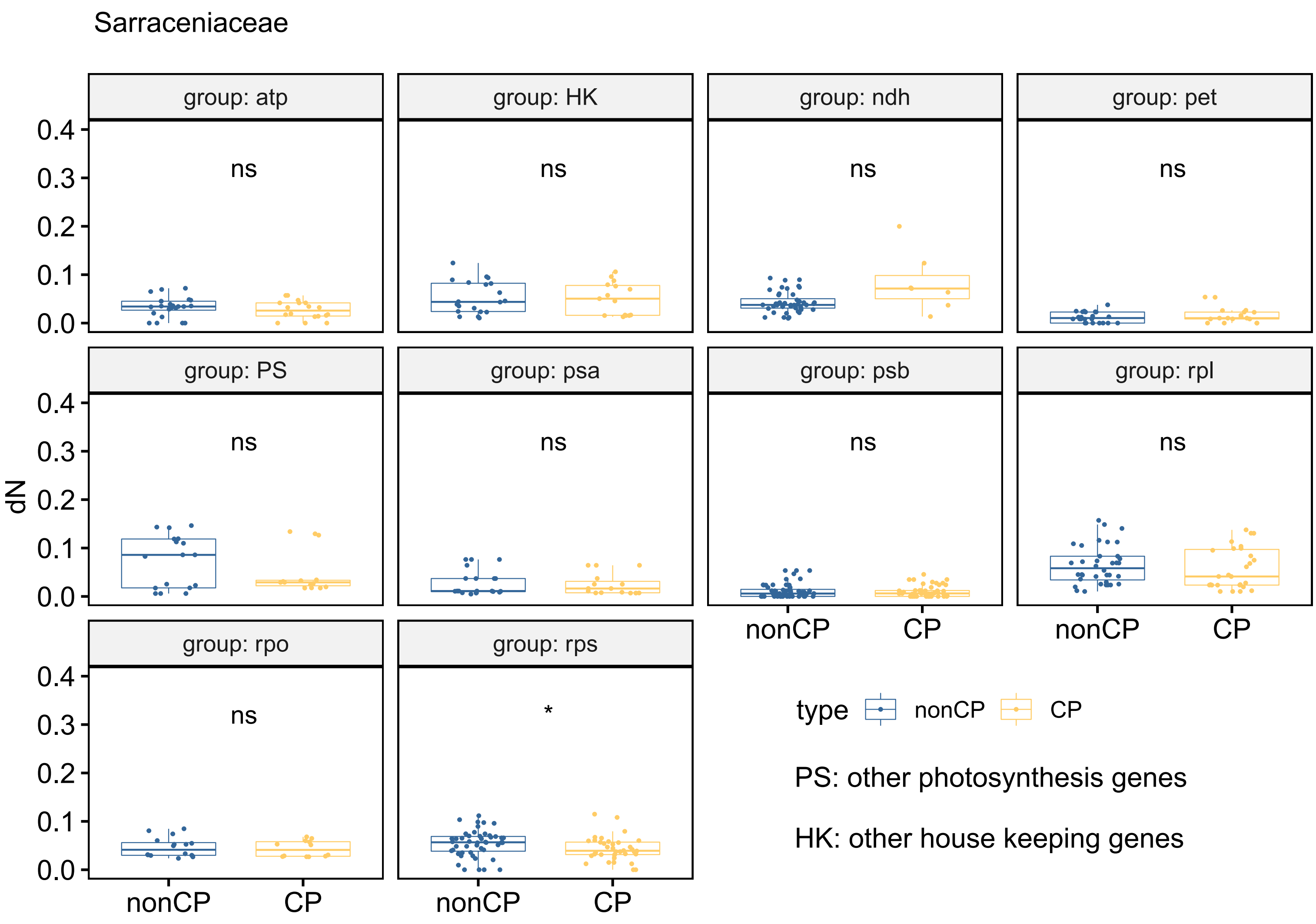

Sarraceniaceae
group: atp
group: HK
group: ndh
group: pet
group: PS
group: psa
group: psb
group: rpl
group: rpo
group: rps
ns
*
dN
0.0
0.1
0.2
0.3
0.4
nonCP
CP
type
nonCP
CP
PS: other photosynthesis genes
HK: other house keeping genes

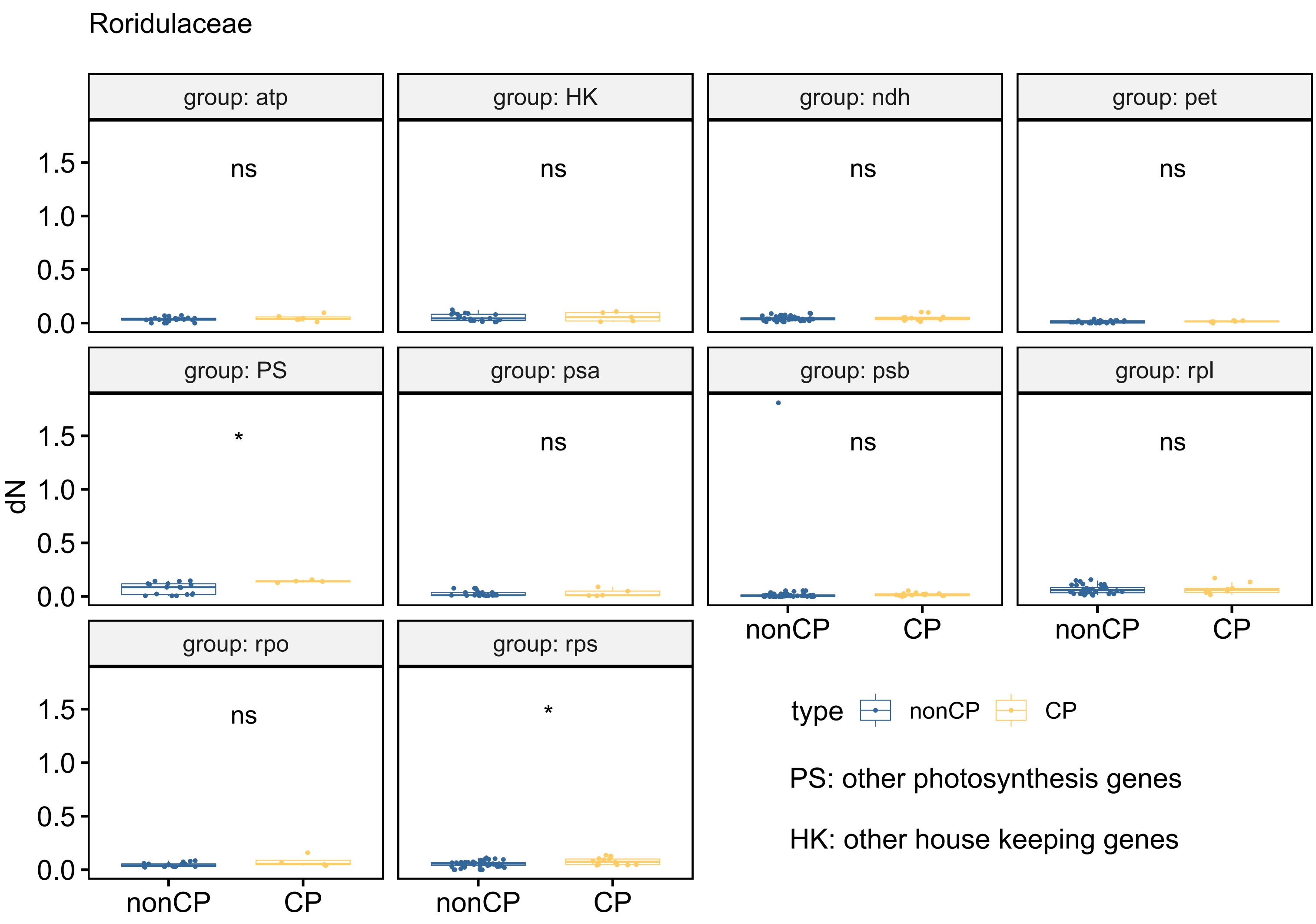

Roridulaceae
group: atp
group: HK
group: ndh
group: pet
group: PS
group: psa
group: psb
group: rpl
group: rpo
group: rps
ns
*
dN
0.0
0.5
1.0
1.5
nonCP
CP
type
PS: other photosynthesis genes
HK: other house keeping genes

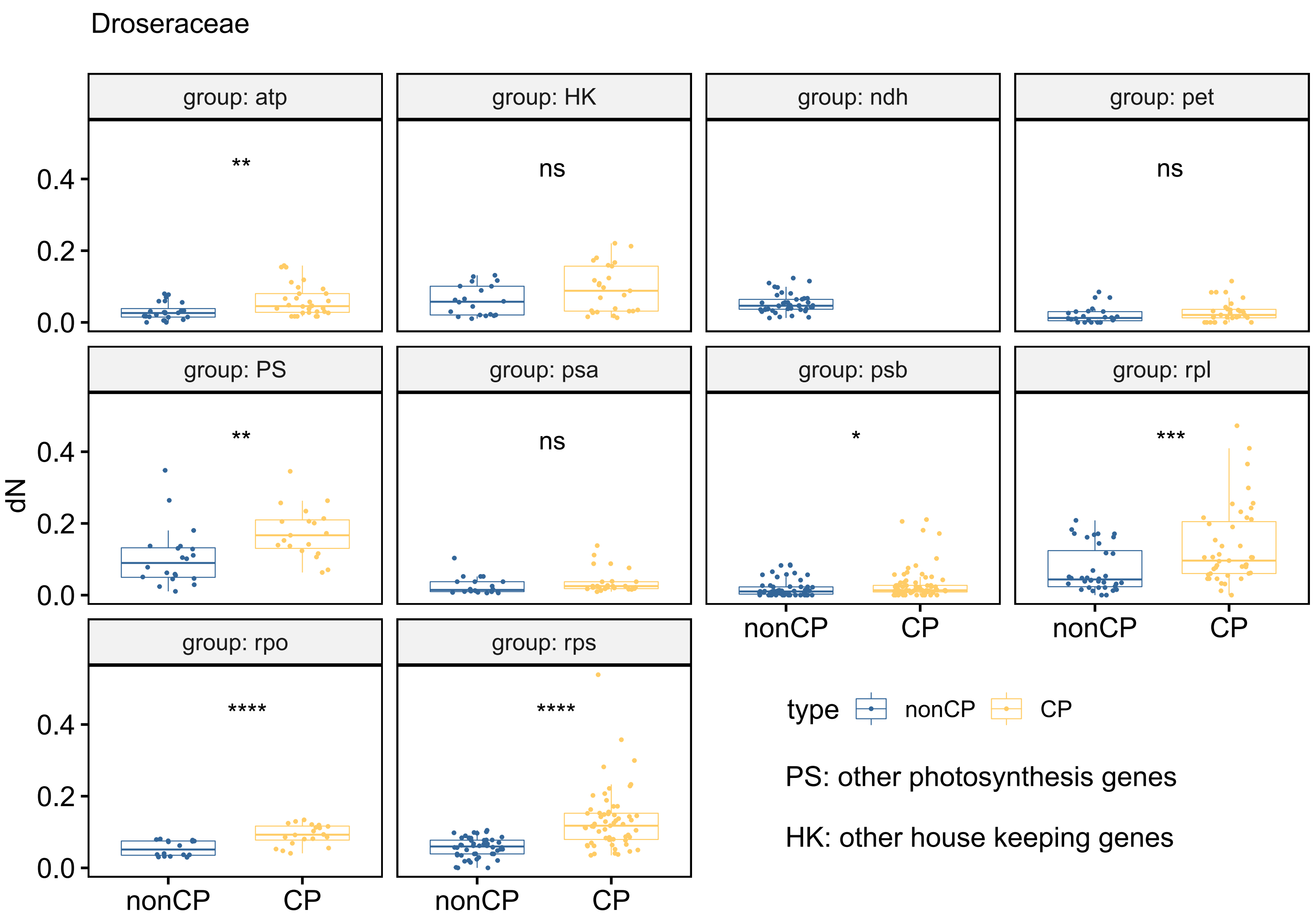
Droseraceae
group: atp
group: HK
group: ndh
group: pet
group: PS
group: psa
group: psb
group: rpl
group: rpo
group: rps
**
ns
ns
**
ns
*
***
****
****
dN
0.0
0.2
0.4
nonCP
CP
type
nonCP
CP
PS: other photosynthesis genes
HK: other house keeping genes

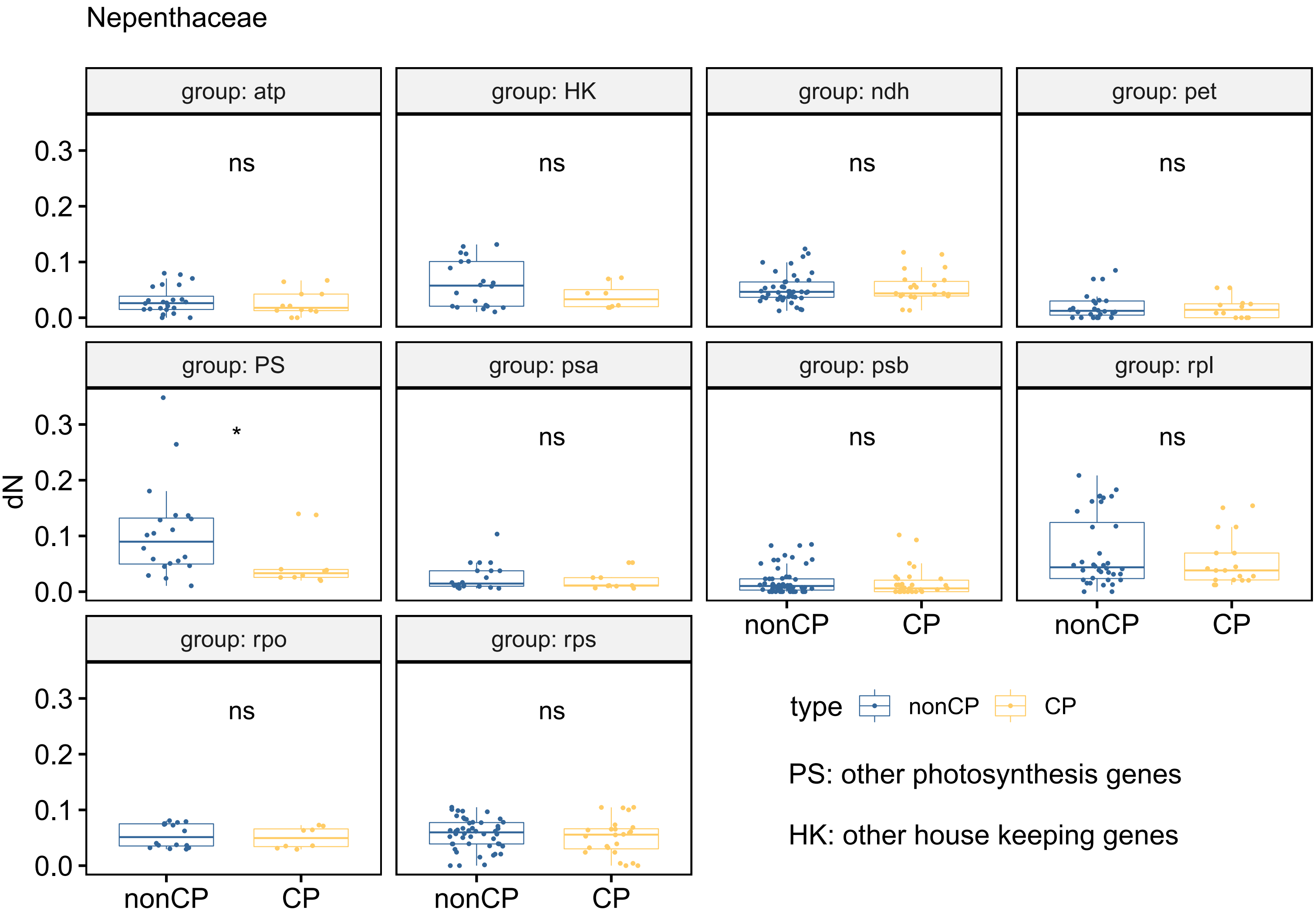

Nepenthaceae
group: atp
group: HK
group: ndh
group: pet
group: PS
group: psa
group: psb
group: rpl
group: rpo
group: rps
ns
*
0.3
0.2
0.1
0.0
dN
nonCP
CP
type
nonCP
CP
PS: other photosynthesis genes
HK: other house keeping genes

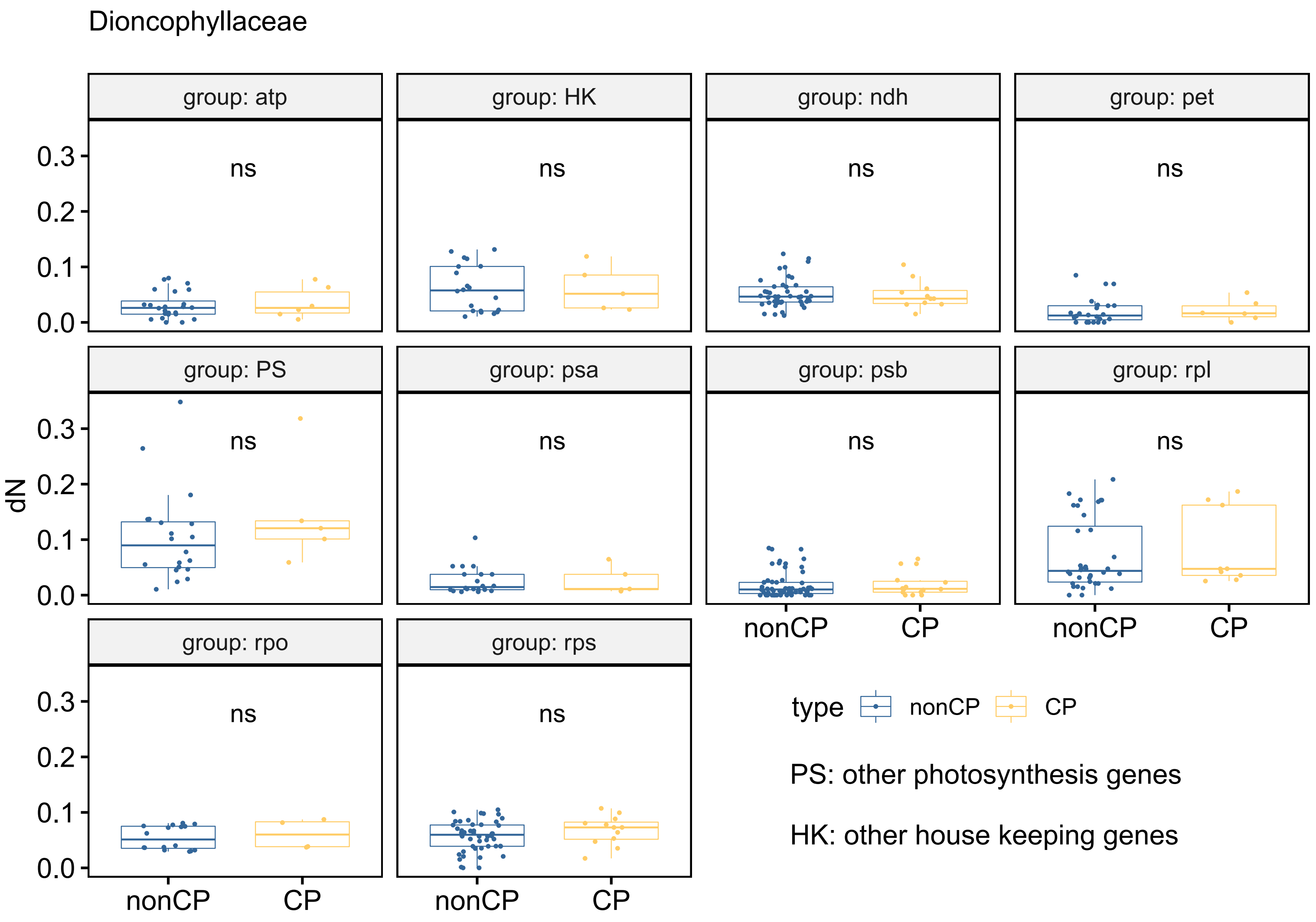
Dioncophyllaceae
group: atp
group: HK
group: ndh
group: pet
group: PS
group: psa
group: psb
group: rpl
group: rpo
group: rps
ns
dN
0.0
0.1
0.2
0.3
nonCP
CP
type
nonCP
CP
PS: other photosynthesis genes
HK: other house keeping genes

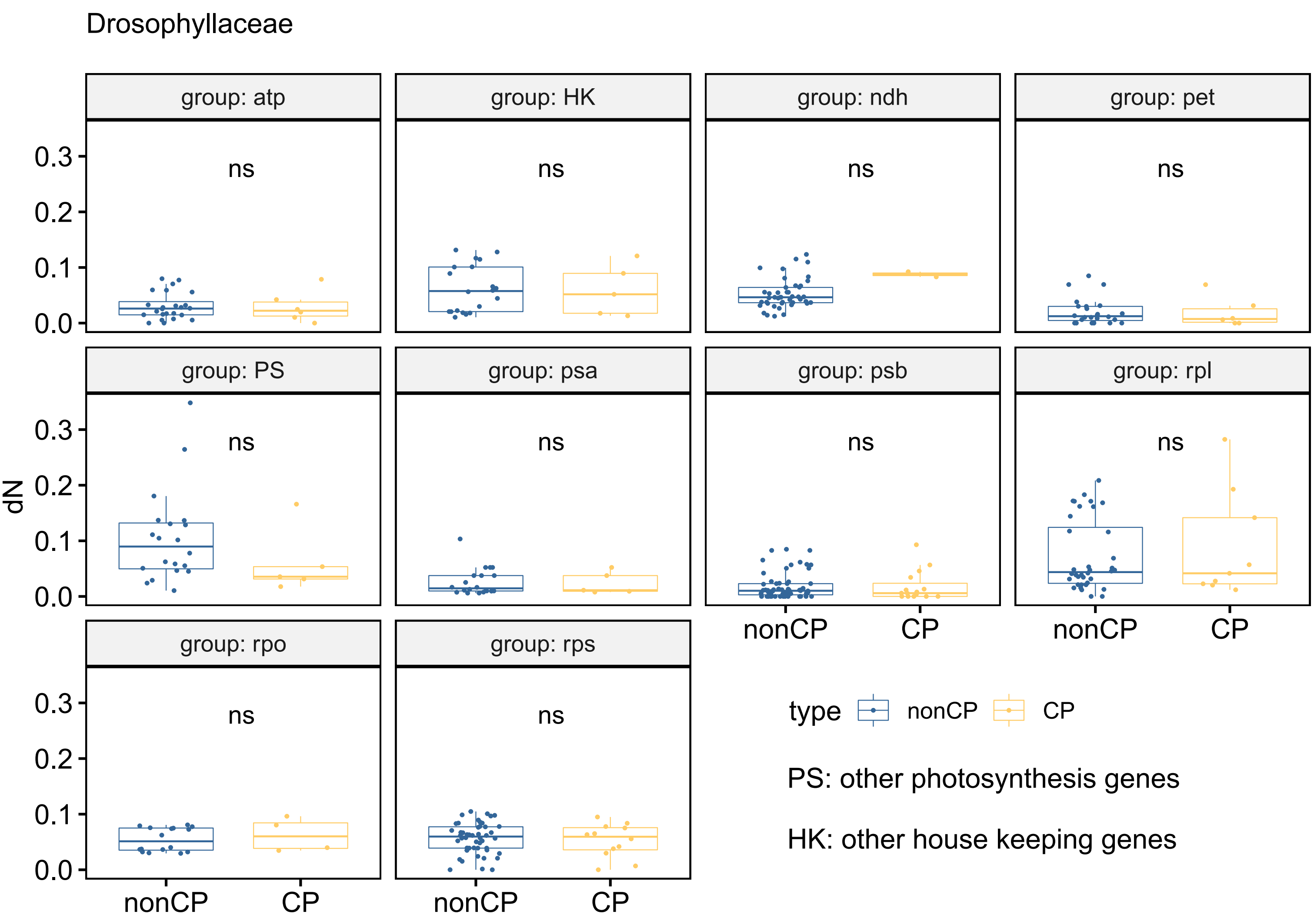

Drosophyllaceae
group: atp
group: HK
group: ndh
group: pet
group: PS
group: psa
group: psb
group: rpl
group: rpo
group: rps
ns
dN
0.0
0.1
0.2
0.3
nonCP
CP
type
nonCP
CP
PS: other photosynthesis genes
HK: other house keeping genes

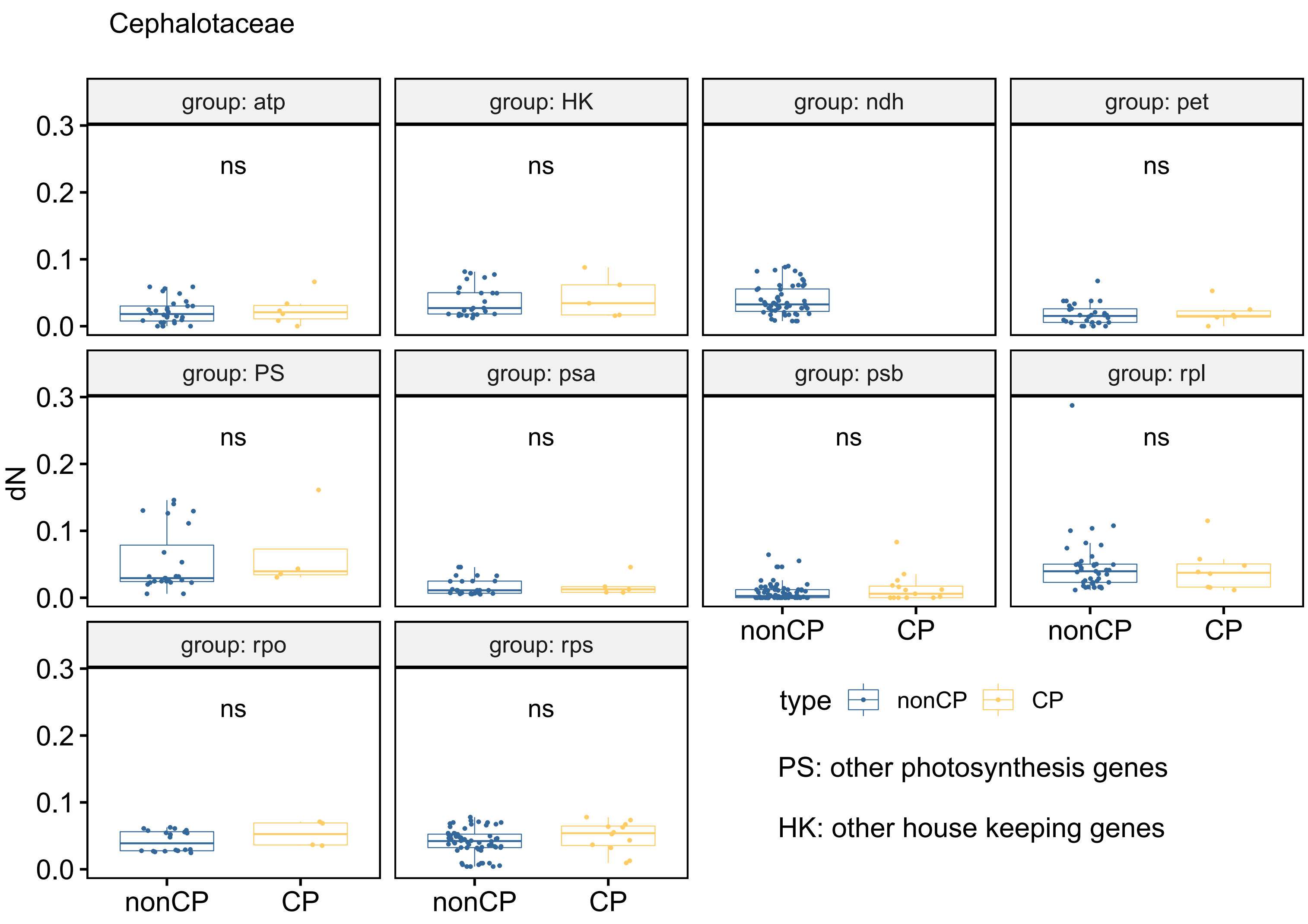
Cephalotaceae
group: atp
group: HK
group: ndh
group: pet
group: PS
group: psa
group: psb
group: rpl
group: rpo
group: rps
ns
dN
0.0
0.1
0.2
0.3
nonCP
CP
type
nonCP
CP
PS: other photosynthesis genes
HK: other house keeping genes

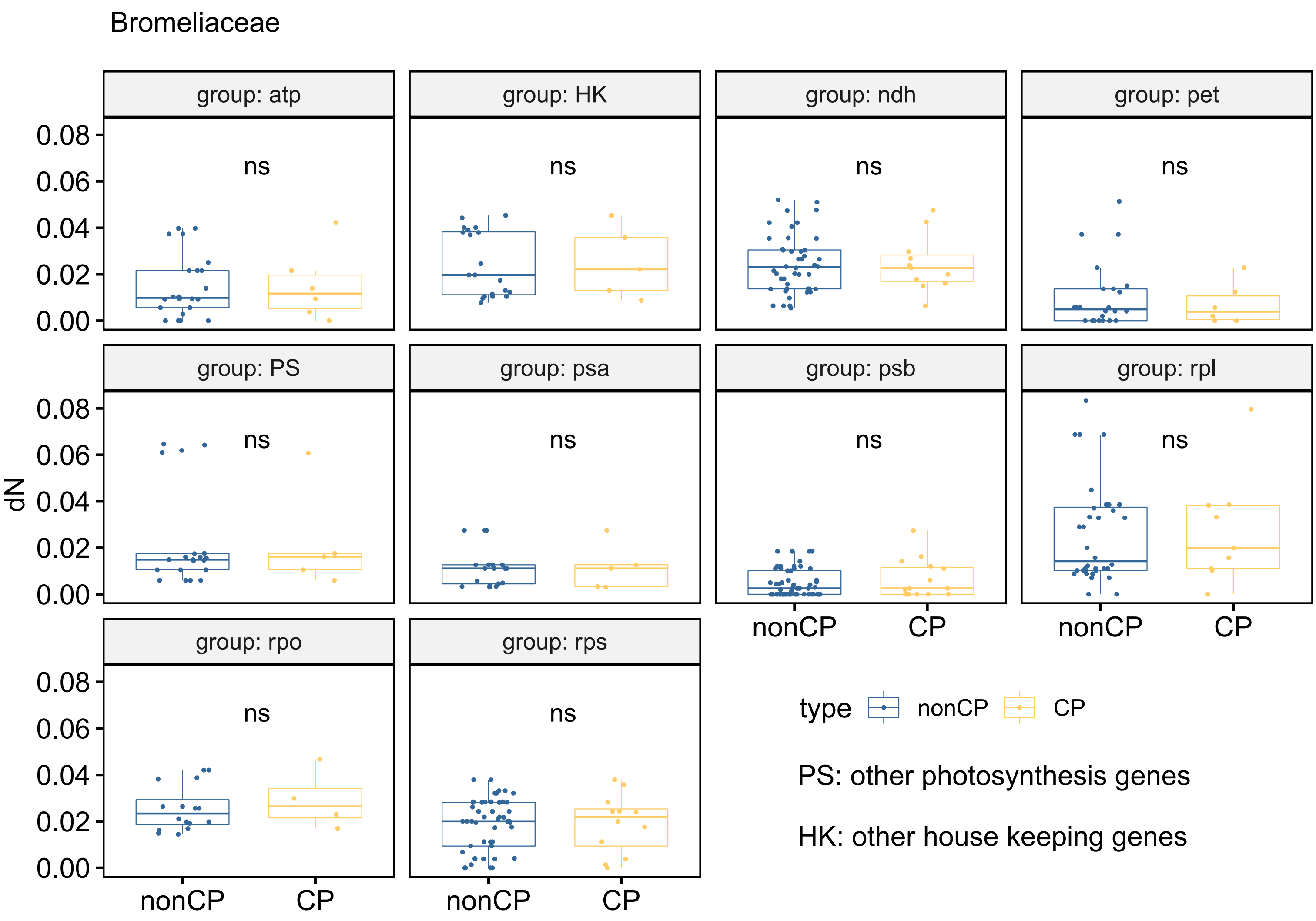

Figure S6. The boxplot illustrates the difference in d_N values between carnivorous and non-carnivorous species for each gene group of each carnivorous and non-carnivorous pair. The PS represents other photosynthesis genes, and HK represents other housekeeping genes. The “*” symbol represents P < 0.05, “**” represents P < 0.01, “***” represents P < 0.001, and “****” represents P < 0.0001.

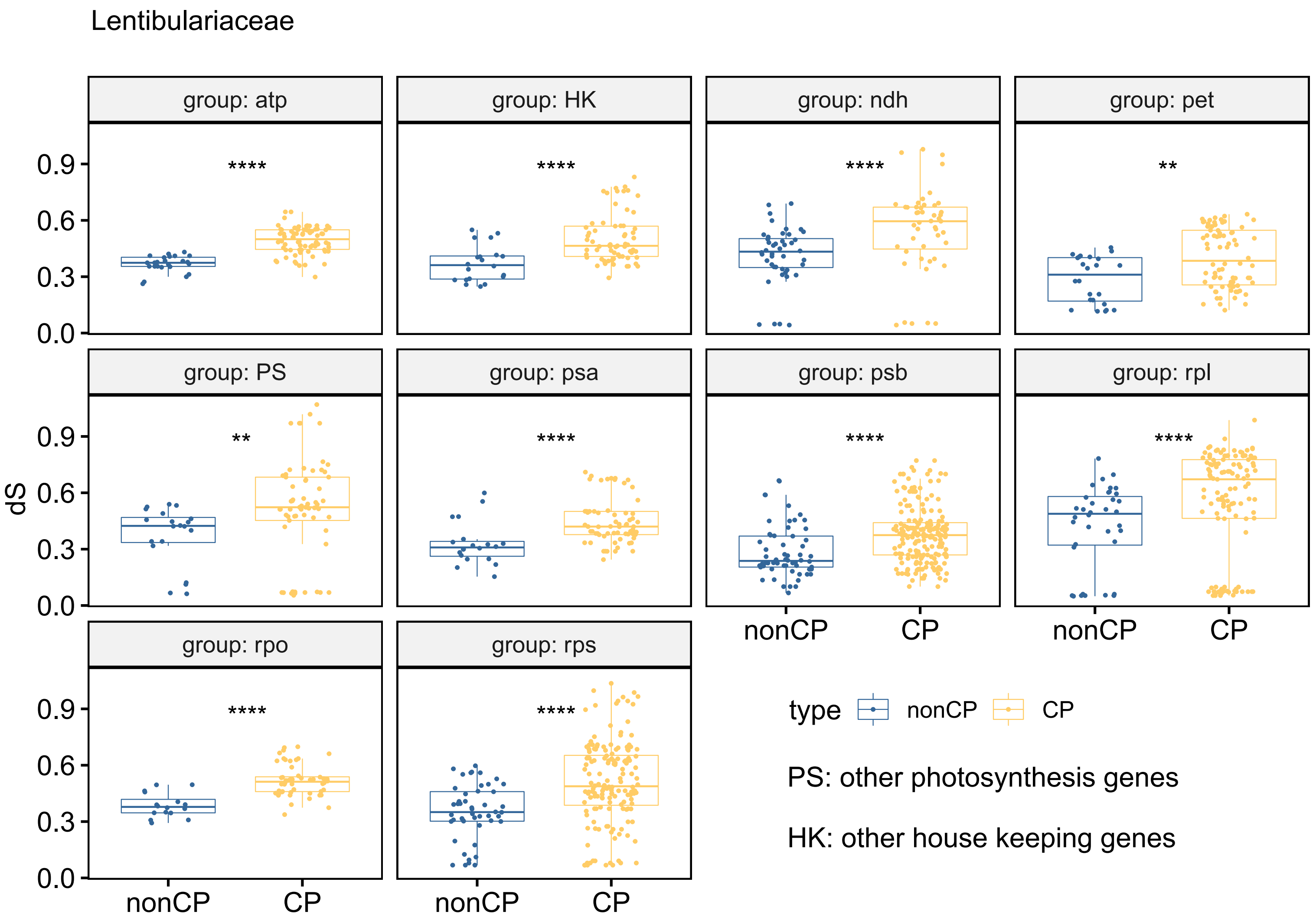
Lentibulariaceae
group: atp
group: HK
group: ndh
group: pet
group: PS
group: psa
group: psb
group: rpl
group: rpo
group: rps
****
**
dS
0.0
0.3
0.6
0.9
nonCP
CP
type
nonCP
CP
PS: other photosynthesis genes
HK: other house keeping genes

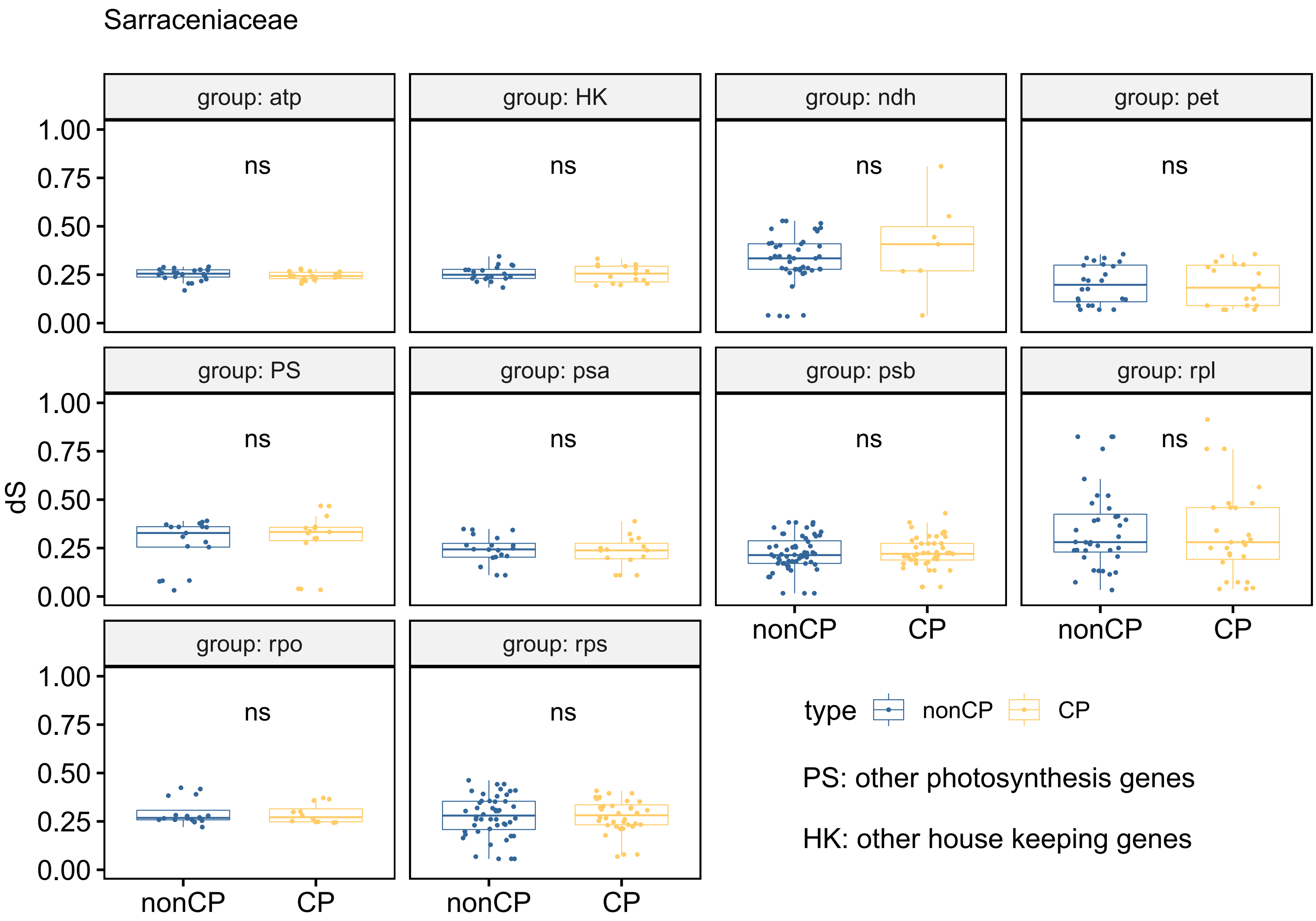

Sarraceniaceae
group: atp
group: HK
group: ndh
group: pet
group: PS
group: psa
group: psb
group: rpl
group: rpo
group: rps
ns
dS
1.00
0.75
0.50
0.25
0.00
nonCP
CP
type
nonCP
CP
PS: other photosynthesis genes
HK: other house keeping genes

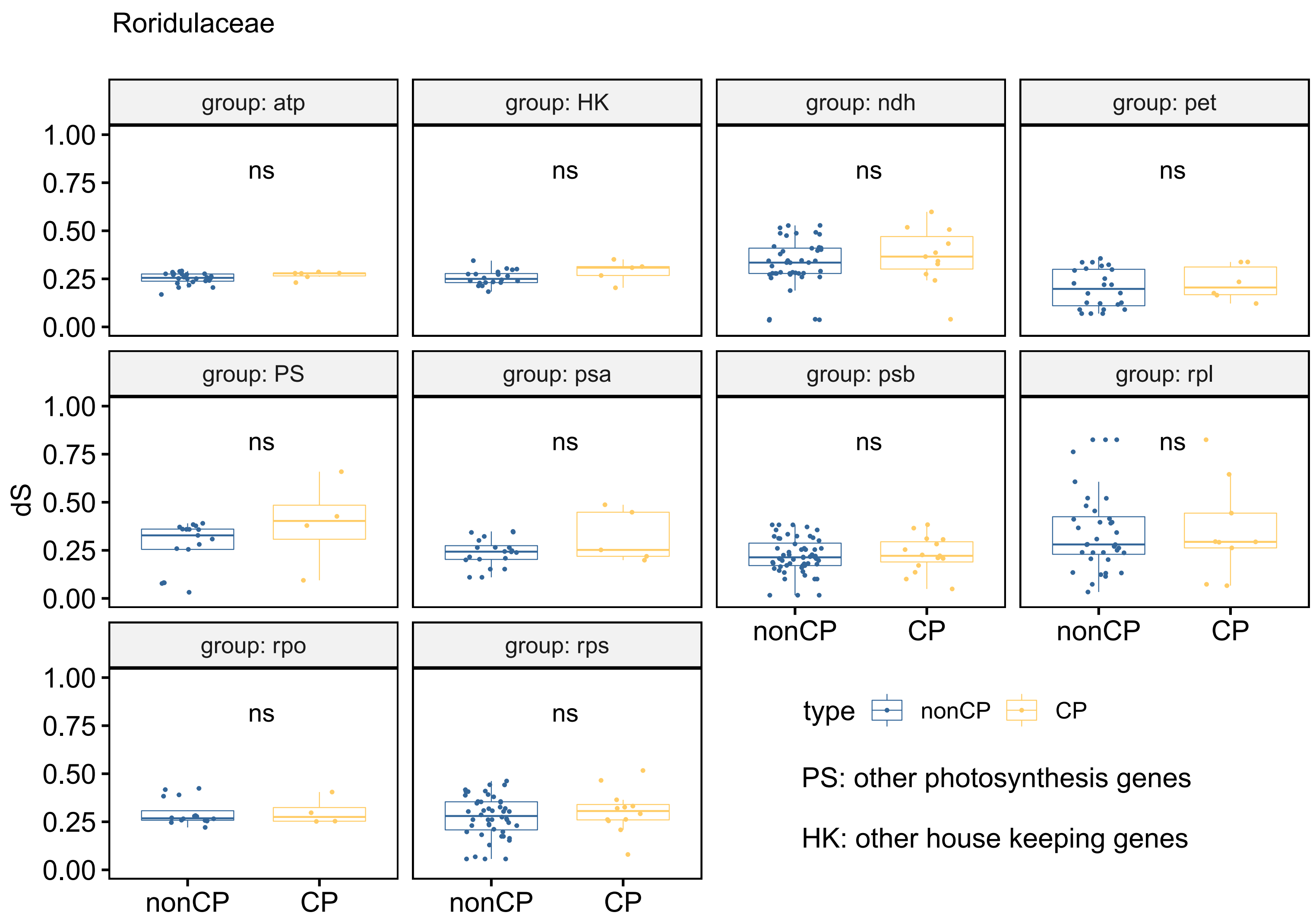
Roridulaceae
group: atp
group: HK
group: ndh
group: pet
group: PS
group: psa
group: psb
group: rpl
group: rpo
group: rps
ns
dS
1.00
0.75
0.50
0.25
0.00
nonCP
CP
type
nonCP
CP
PS: other photosynthesis genes
HK: other house keeping genes

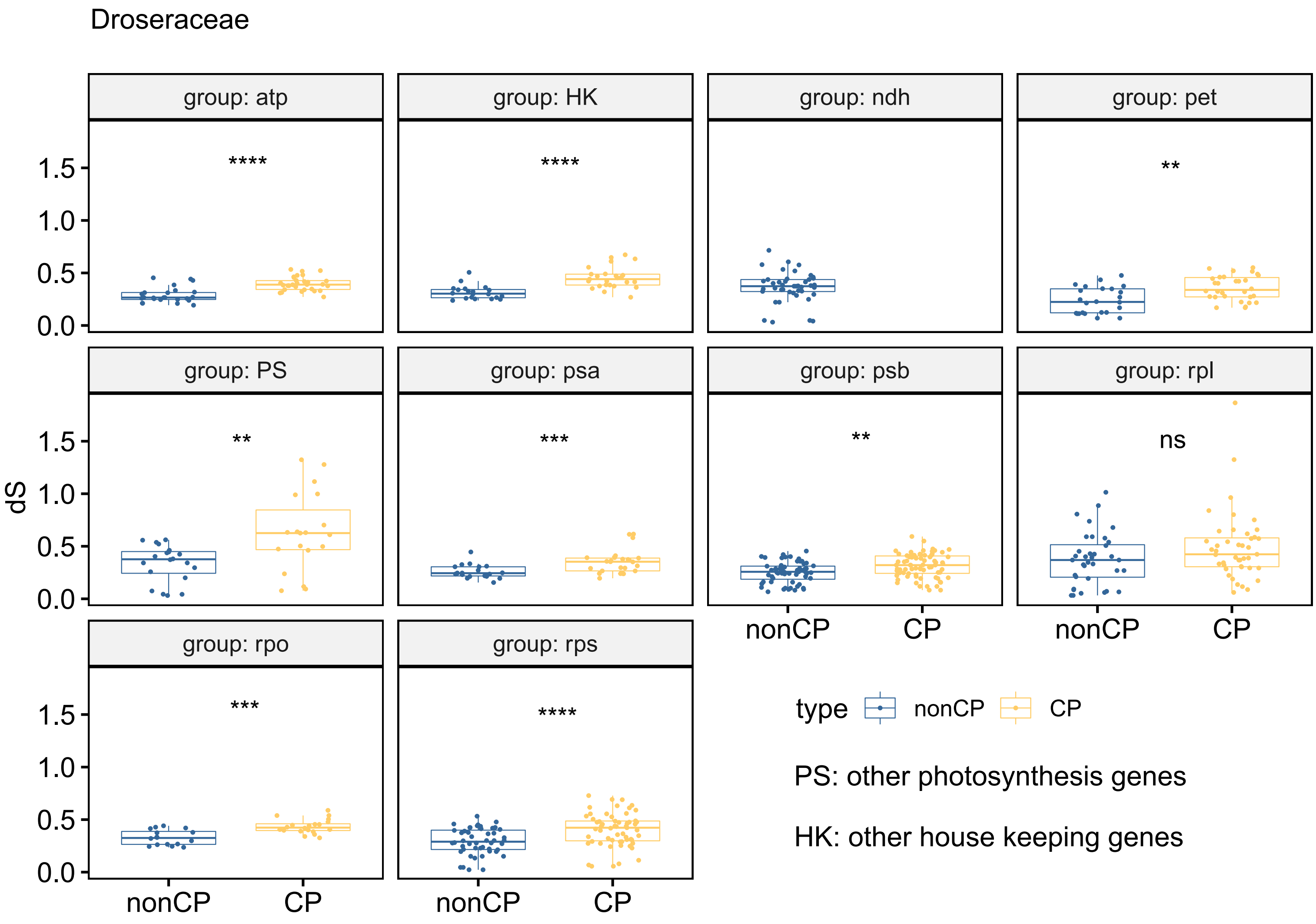

Droseraceae
group: atp
group: HK
group: ndh
group: pet
group: PS
group: psa
group: psb
group: rpl
group: rpo
group: rps
****
****
**
**
***
**
ns
***
****
dS
0.0
0.5
1.0
1.5
nonCP
CP
type
nonCP
CP
PS: other photosynthesis genes
HK: other house keeping genes

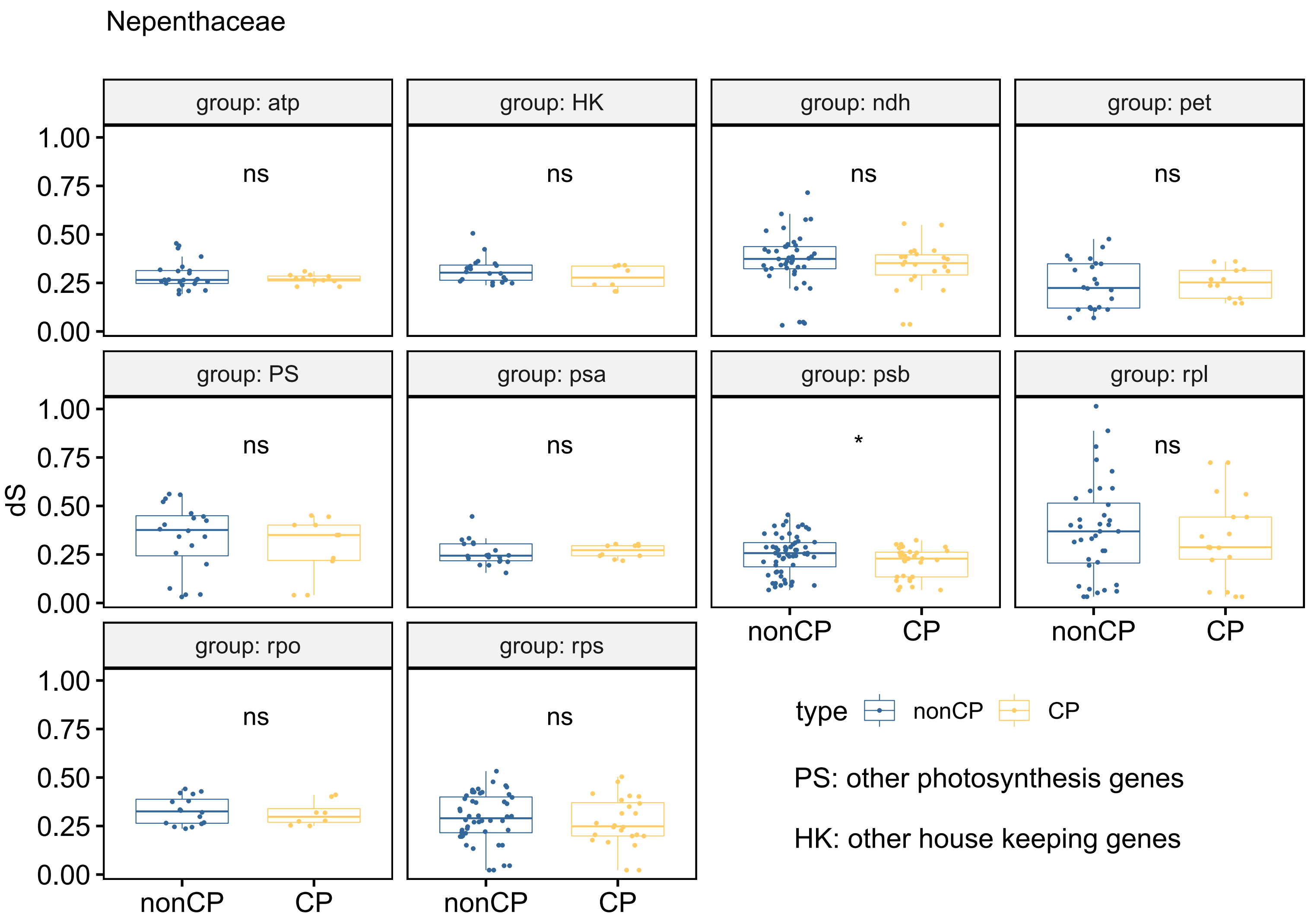

Nepenthaceae
group: atp
group: HK
group: ndh
group: pet
group: PS
group: psa
group: psb
group: rpl
group: rpo
group: rps
ns
*
dS
1.00
0.75
0.50
0.25
0.00
nonCP
CP
type
nonCP
CP
PS: other photosynthesis genes
HK: other house keeping genes

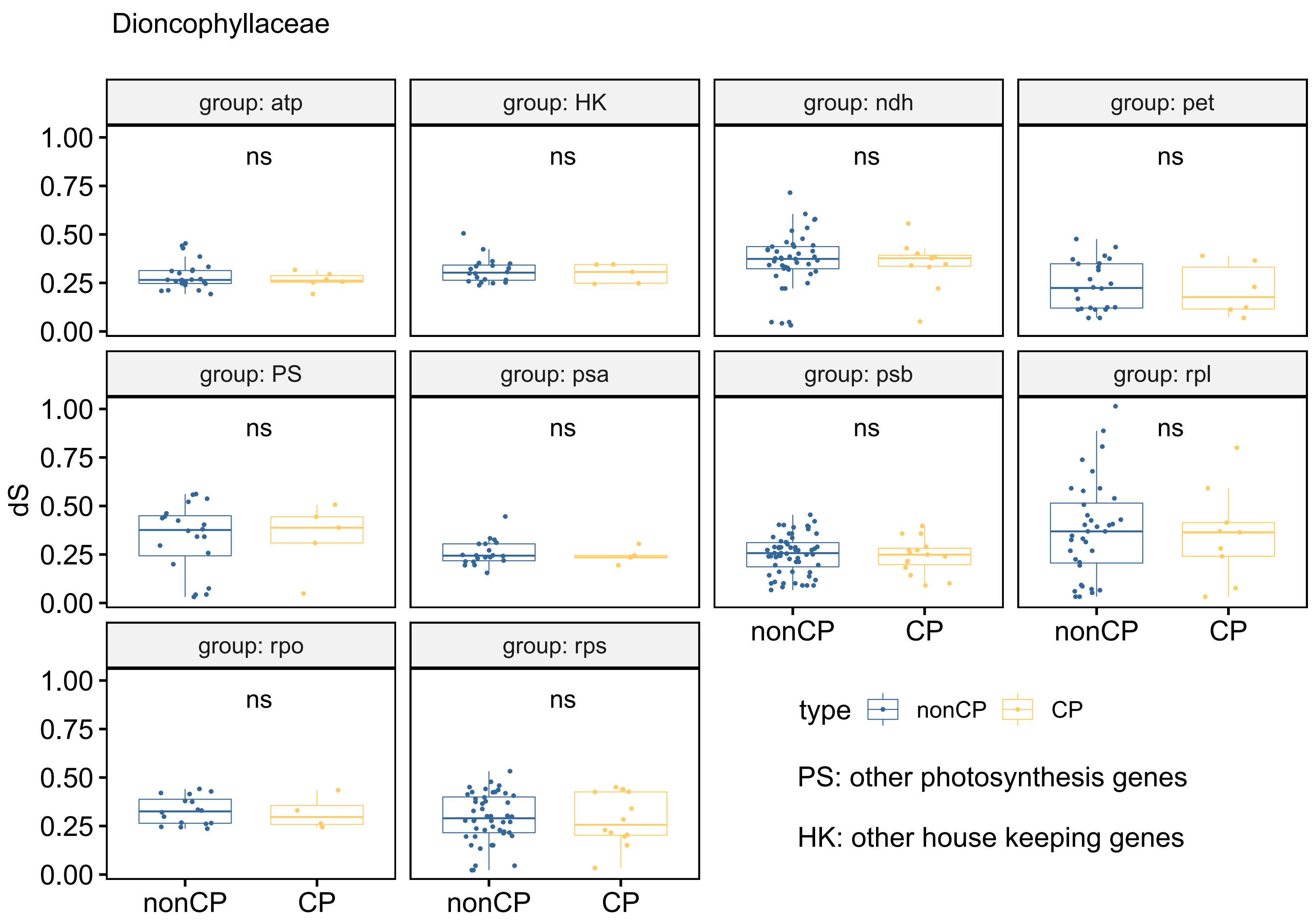

Dioncophyllaceae
group: atp
group: HK
group: ndh
group: pet
group: PS
group: psa
group: psb
group: rpl
group: rpo
group: rps
ns
dS
1.00
0.75
0.50
0.25
0.00
nonCP
CP
type
nonCP
CP
PS: other photosynthesis genes
HK: other house keeping genes

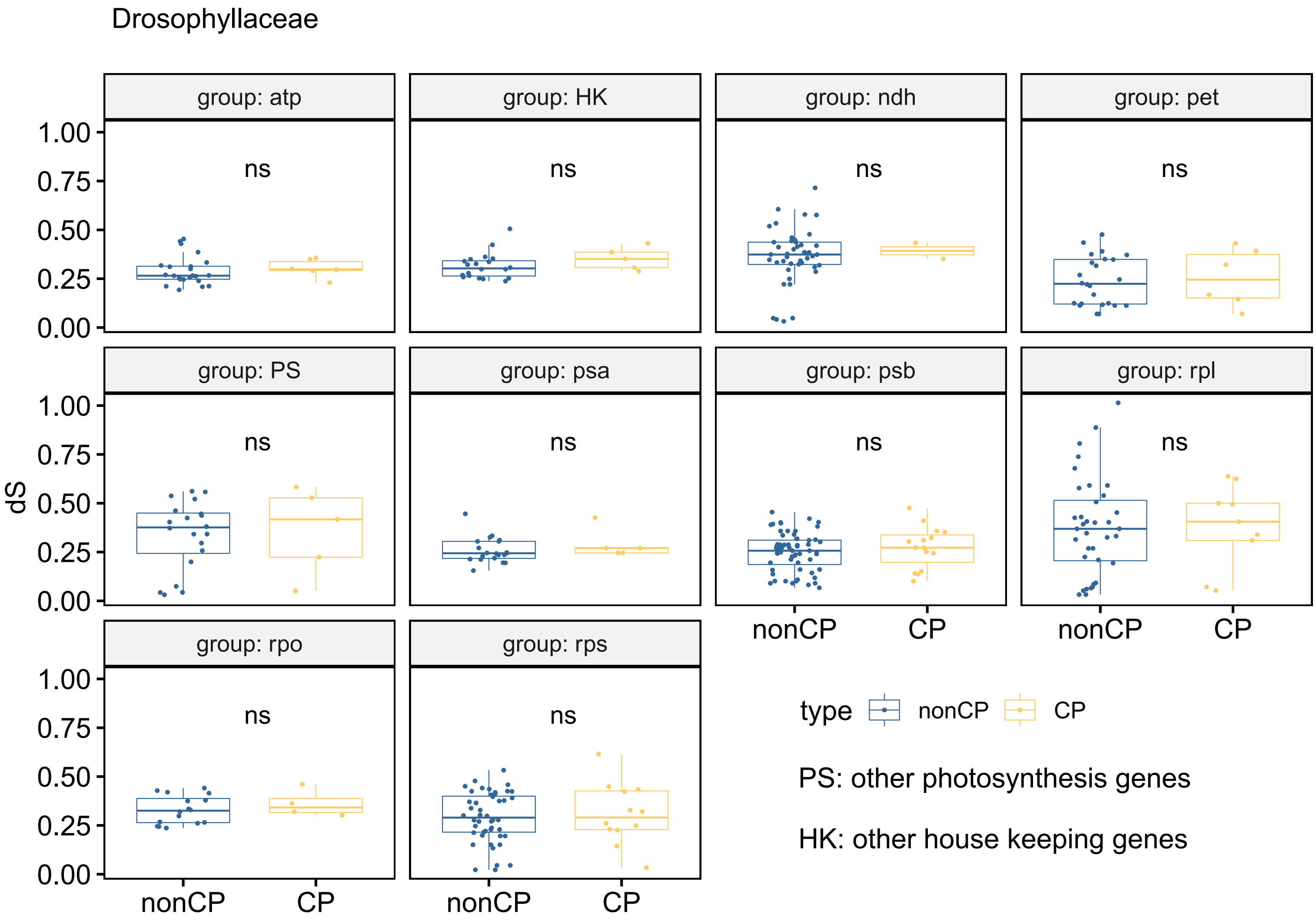

Drosophyllaceae
group: atp
group: HK
group: ndh
group: pet
group: PS
group: psa
group: psb
group: rpl
group: rpo
group: rps
ns
dS
1.00
0.75
0.50
0.25
0.00
nonCP
CP
type
nonCP
CP
PS: other photosynthesis genes
HK: other house keeping genes

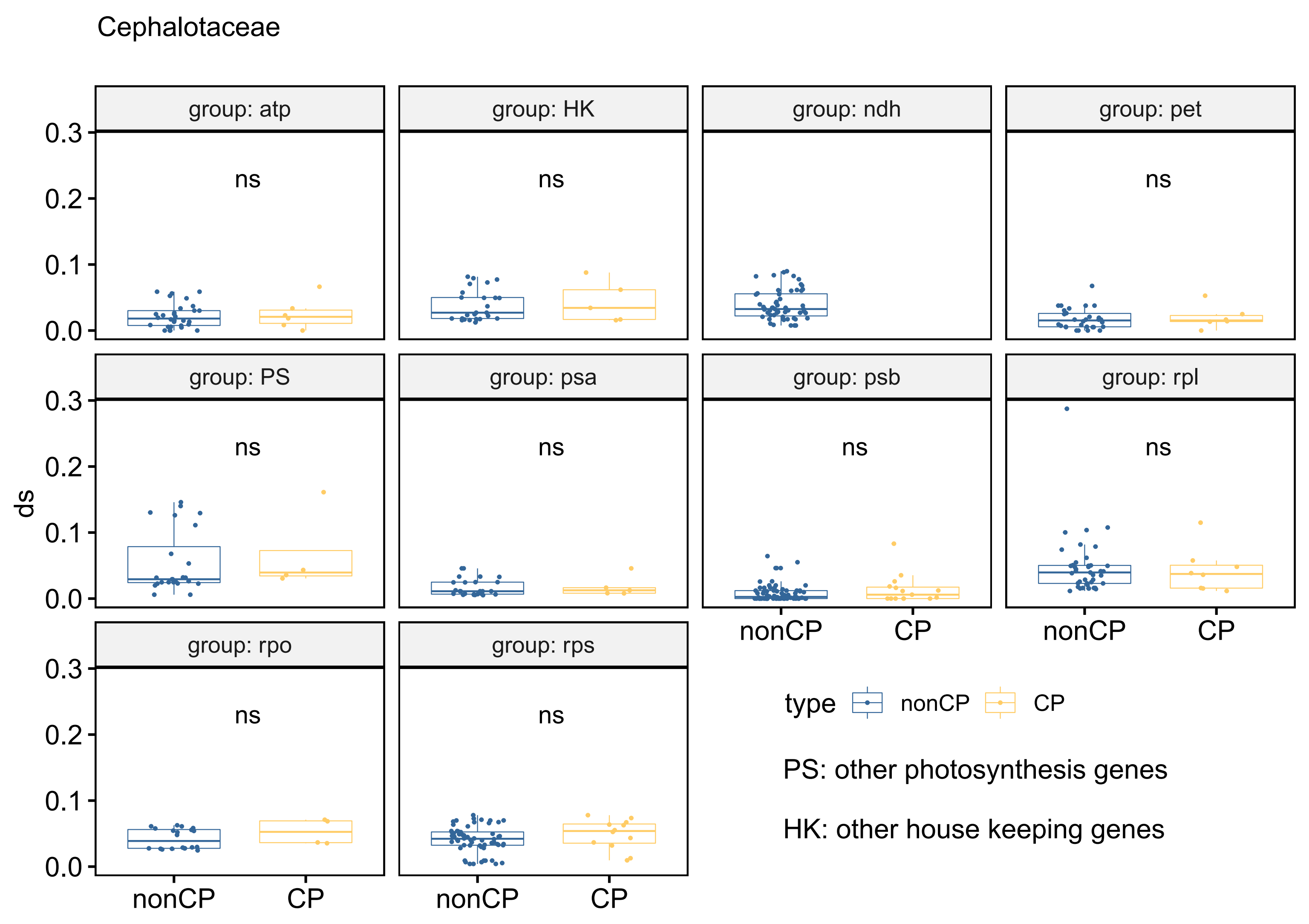

Cephalotaceae
group: atp
group: HK
group: ndh
group: pet
group: PS
group: psa
group: psb
group: rpl
group: rpo
group: rps
ns
ds
0.0
0.1
0.2
0.3
nonCP
CP
type
nonCP
CP
PS: other photosynthesis genes
HK: other house keeping genes

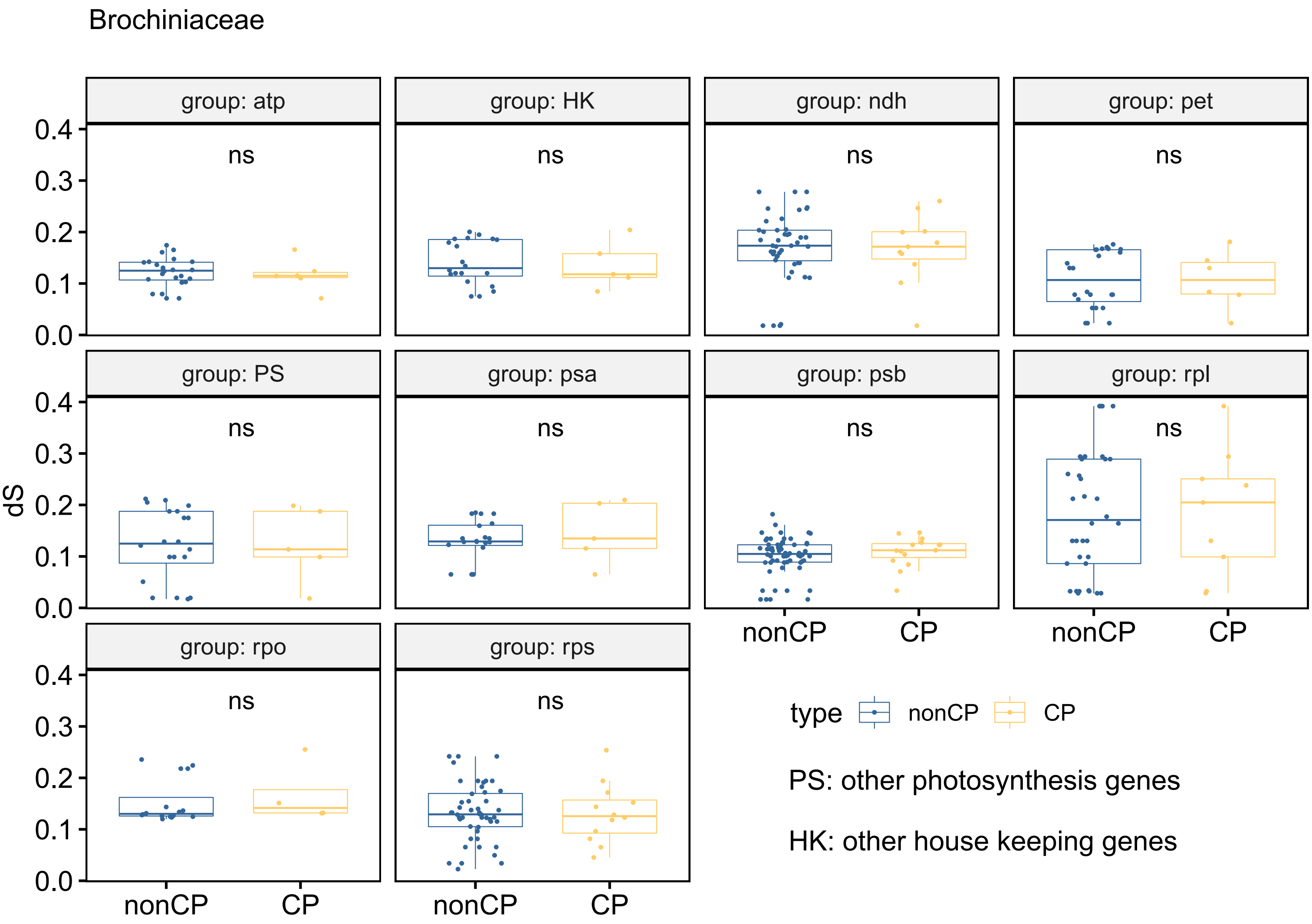

Figure S7. The boxplot illustrates the difference in *d*s values between carnivorous and non-carnivorous species for each gene groups of each carnivorous and non-carnivorous pair. The PS represents other photosynthesis genes, and HK represents other housekeeping genes. The “*” symbol represents P < 0.05, “**” represents P < 0.01, “***” represents P < 0.001, and “****” represents P < 0.0001.
